# Rafoxanide disrupts mitochondrial homeostasis through VDAC1 modulation in colorectal cancer cells

**DOI:** 10.1038/s41420-026-02986-3

**Published:** 2026-03-05

**Authors:** Lorenzo Tomassini, Teresa Pacifico, Mattia Alberto Serra, Eduardo Maria Sommella, Manolo Sambucci, Giuseppe Sigismondo Sica, Luca Savino, Sara Vitale, Angela Ortenzi, Livia Biancone, Luca Battistini, Giovanna Borsellino, Ivan Monteleone, Vincenzo Barnaba, Micol Eleonora Fiori, Giovanni Monteleone, Carmine Stolfi, Federica Laudisi

**Affiliations:** 1 Department of Systems Medicine, University of Rome Tor Vergata, Rome, Italy; 2 Department of Pharmacy, University of Salerno, Fisciano, Salerno Italy; 3 Neuroimmunology Unit, Santa Lucia Foundation IRCCS, Rome, Italy; 4 Department of Surgery, University of Rome Tor Vergata, Rome, Italy; 5 Department of Integrated Care Processes, University of Rome Tor Vergata, Rome, Italy; 6 Department of Oncology and Molecular Medicine, Istituto Superiore di Sanità, Rome, Italy; 7 Gastroenterology Unit, Policlinico Universitario Tor Vergata, Rome, Italy; 8 Department of Biomedicine and Prevention, University of Rome Tor Vergata, Rome, Italy; 9 Istituto Pasteur Italia and Sapienza Università di Roma, Rome, Italy

**Keywords:** Colon cancer, Cancer metabolism

## Abstract

Functional mitochondria are essential for cancer cells, as they sustain oxidative phosphorylation, metabolic flexibility and survival. Targeting mitochondrial homeostasis has therefore emerged as a promising strategy to sensitize cancer cells to cell death. Rafoxanide is a halogenated salicylanilide originally developed as a veterinary anthelmintic and described to exert mitochondrial uncoupling activity in parasitic organisms. Although rafoxanide has been shown to exert potent antitumor activity against colorectal cancer (CRC), the mechanisms underlying this effect remain incompletely understood. Here, we investigated the impact of rafoxanide on mitochondrial function and stress responses in CRC cells. Rafoxanide rapidly impaired mitochondrial respiration, reducing basal and maximal oxygen consumption and ATP-related respiration, and induced a progressive but reversible dissipation of mitochondrial membrane potential. Integrated transcriptomic, proteomic, and metabolomic analyses revealed that prolonged rafoxanide exposure resulted in sustained mitochondrial dysfunction, failure of metabolic adaptation, and release of cytochrome *c* from the mitochondria into the cytosol. Mechanistically, rafoxanide inhibited mitochondrial respiratory chain complexes I and III, leading to a rapid increase in total cellular reactive oxygen species. This redox imbalance promoted voltage-dependent anion channel (VDAC1) oligomerization and mitochondrial outer membrane permeabilization. Notably, mitochondrial superoxide production was reduced at later time points, consistent with the loss of mitochondrial membrane potential rather than the absence of a cellular oxidative stress response. Finally, proteomic analysis of colonic lesions from a murine model of sporadic CRC, as well as human CRC explants and intestinal organoids, confirmed that rafoxanide consistently alters mitochondrial protein expression and function across in vitro, in vivo, and ex vivo systems. In conclusion, our results identify rafoxanide as a modulator of mitochondrial homeostasis that induces redox-dependent VDAC1 activation and progressive mitochondrial dysfunction in CRC cells, providing mechanistic insight into its antitumor activity and supporting further exploration of mitochondrial stress modulation as a therapeutic strategy in CRC.

## Introduction

Mitochondria are essential organelles that support a wide array of vital cellular processes, including ATP generation through oxidative phosphorylation (OXPHOS), maintenance of redox balance, synthesis of key biosynthetic precursors in the cytosol, and the orchestration of programmed cell death [1]. These functions are made possible by their unique architecture, which comprises an outer mitochondrial membrane (OMM) enclosing the organelle and a highly invaginated inner mitochondrial membrane (IMM) forming cristae that maximize the surface area available for electron transport chain (ETC) complexes and ATP synthase [1]. Mitochondrial membrane permeability is under strict control. While the OMM is relatively permeable to small solutes, the IMM is highly selective to protect the electrochemical gradient essential for ATP production. Voltage-Dependent Anion Channel 1 (VDAC1) is the most abundant pore-forming protein in the OMM and a key regulator of its permeability [2, 3]. Indeed, VDAC1 controls the flux of metabolites (such as ATP, ADP, pyruvate, and inorganic ions) between mitochondria and the cytosol, acting in combination with components of the mitochondrial permeability transition pore (mPTP). In addition to this metabolic gatekeeping role, VDAC1 is involved in the regulation of multiple regulatory pathways. Indeed, VDAC1 closure upon binding to tubulin C-terminal tails reprograms cellular bioenergetics by reducing metabolite exchange [4]. VDAC1 can also interact with hexokinase to anchor glycolysis to the mitochondrial surface, stabilizing mitochondrial membrane potential and contributing to the Warburg effect [5]. Through these activities, VDAC1 helps regulate ROS production and mitochondrial homeostasis. In apoptotic or cell stress conditions, VDAC1 can oligomerize or coordinate with pro-apoptotic members of the Bcl-2 family, such as Bax and Bak, promoting the release of cytochrome *c* and activating caspases [2, 6]. Thus, VDAC1 is a major determinant of mitochondrial function and cell fate at the intersection of metabolism and cell death.

The tight regulation of mitochondrial activity is particularly relevant to cancer biology. Although mutations in mitochondrial DNA (mtDNA) are frequently found in tumors [7], leading to the initial belief that cancer cells rely less on mitochondrial metabolism, mounting evidence demonstrates that fully functional mitochondria are essential for cancer cell proliferation, survival, and metastatic progression [8, 9]. Indeed, functional mitochondria supply rapidly dividing cells with ATP, NADH, and biosynthetic intermediates, including pyrimidines, lipids, and tricarboxylic acid (TCA) cycle-derived metabolites. Interfering with these processes often results in mitochondrial dysfunction and cell death, highlighting mitochondrial metabolism as a therapeutic vulnerability. Accordingly, compounds that impair mitochondrial activity or alter bioenergetic flux have emerged as promising adjuvants or sensitizers in cancer therapy [10, 11].

Among such compounds, mitochondrial uncouplers represent a class of molecules that disrupt OXPHOS by collapsing the proton motive force, thereby disconnecting ETC activity from ATP synthesis. Although classical uncoupling is typically associated with loss of mitochondrial membrane potential, reduced ATP synthesis, and metabolic stress, ultimately leading to cell death [12–14], accumulating evidence indicates that the biological consequences of uncoupling in mammalian cells are highly context-dependent. In particular, partial or transient uncoupling can modulate mitochondrial redox balance, electron transport chain activity, and stress signaling pathways without necessarily causing an immediate bioenergetic collapse or overt cytotoxicity.

Several compounds originally developed as mitochondrial uncouplers in non-mammalian systems, including halogenated salicylanilides, have recently attracted interest in oncology due to their ability to interfere with cancer cell metabolism, stress adaptation, and survival. In colorectal cancer (CRC), salicylanilide derivatives such as niclosamide ethanolamine (NEN) and oxyclozanide have been shown to impair tumor growth and migration, yet their antitumor activity cannot be fully explained by a simple uncoupling model. Rather, these molecules appear to reprogram mitochondrial function and cellular stress responses through mechanisms that remain incompletely defined.

Rafoxanide is a halogenated salicylanilide originally developed as a veterinary anthelmintic and described to exert mitochondrial uncoupling activity in helminths [15]. However, increasing evidence indicates that salicylanilide compounds can exert diverse and context-dependent biological effects in mammalian cells, extending beyond canonical uncoupling mechanisms. We previously reported that rafoxanide selectively inhibits CRC cell proliferation, while sparing normal colonic epithelial cells, both in vitro and in vivo, including patient-derived CRC explants. These effects were associated with the activation of the endoplasmic reticulum (ER) stress response followed by caspase-dependent apoptosis [16]. Moreover, rafoxanide was shown to suppress STAT3/NF-κB signaling at multiple levels within the CRC microenvironment and to reduce inflammation-associated colon tumorigenesis in vivo [17]. Together, these findings suggest that the drug may interfere with fundamental stress-adaptation pathways in cancer cells, rather than acting as a non-specific cytotoxic agent.

Despite its classification as an uncoupler in parasitic organisms, it remains unclear whether rafoxanide induces a comparable bioenergetic collapse in mammalian tumor cells or instead elicits a more nuanced modulation of mitochondrial function. In particular, the contribution of mitochondrial outer membrane regulators, such as VDAC1, to rafoxanide-induced cellular stress and cell death has not been explored. Whether mitochondrial dysfunction represents a primary trigger or a downstream consequence of rafoxanide-mediated ER stress and carcinogenetic pathway inhibition remains an open and mechanistically relevant question. In this study, we investigated the impact of rafoxanide on mitochondrial homeostasis in CRC cells, with particular focus on VDAC1 regulation, mitochondrial membrane potential, redox balance, and the execution of programmed cell death.

## Results

### Rafoxanide impairs mitochondrial oxygen consumption and decreases mitochondrial membrane potential in CRC cells

**Fig. 1:**
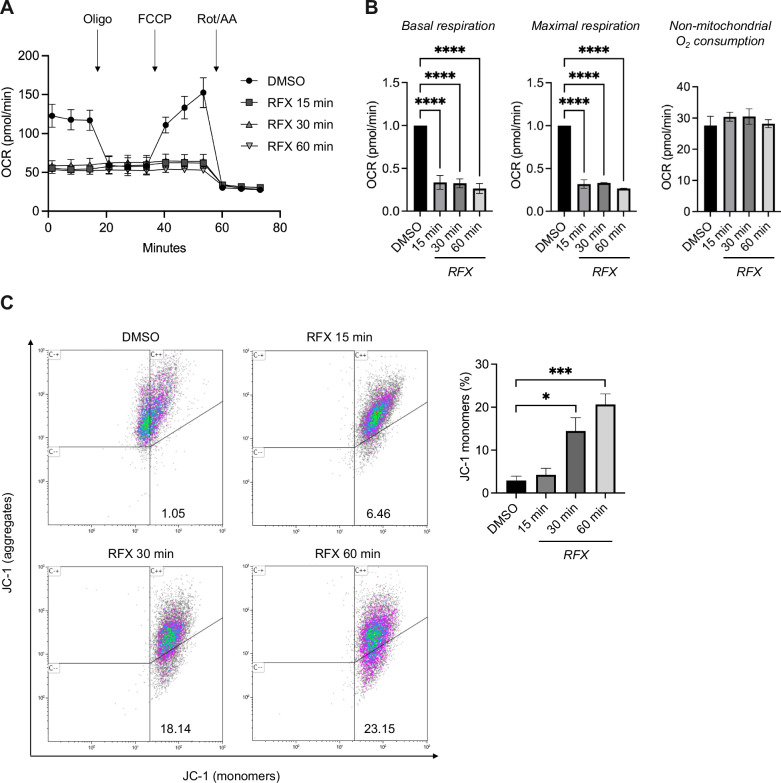
Rafoxanide impairs mitochondrial respiration and membrane potential in CRC cells. **A** Representative bioenergetic profile obtained by measuring oxygen consumption rate (OCR) of HCT116 cells after treatment with either rafoxanide (RFX, 2.5 μM) or vehicle (DMSO) for 15, 30, and 60 min. **B** The histograms show indices of mitochondrial respiratory function. Data are expressed as fold change of mean values ± SEM with the DMSO control set to 1 of 3 independent experiments. Differences among groups were compared using one-way analysis of variance (ANOVA) followed by the Tukey test (****P ≤ .0001). **C** *Left panel:* representative dot plots from flow cytometry analysis showing JC-1 fluorescence in HCT116 cells treated with rafoxanide (RFX, 2.5 μM) or DMSO (vehicle) for 15, 30, and 60 min. JC-1 aggregates indicate polarized mitochondria, whereas JC-1 monomers indicate mitochondrial membrane depolarization. Numbers indicate the percentage of cells in the designated quadrants. Right panel: bar plots showing the fraction of depolarized mitochondria in HCT116 cells treated as indicated in panel (**C**). Values are mean ± SEM of 3 independent experiments. Differences among groups were compared using one-way ANOVA followed by Tukey’s post hoc test (*P ≤ .05, **P ≤ .01, ***P ≤ .001).

To evaluate whether rafoxanide impairs mitochondrial activity in CRC cells, we quantified mitochondrial oxygen consumption rate (OCR) in HCT116 and DLD1 cell lines at different time points (15, 30, and 60 min) upon stimulation with rafoxanide (2.5 µM). Rafoxanide inhibited the overall mitochondrial OCR in HCT116 (Fig. 1A) and DLD1 cells (not shown) already after 15 min post-treatment. In particular, basal and maximal respiration were downregulated by rafoxanide compared to control (DMSO) (Fig. 1B), while non-mitochondrial oxygen consumption rate remained unaltered (Fig. 1B), suggesting a specific impairment of mitochondrial activity by the anthelmintic drug. Rafoxanide-treated HCT116 cells also underwent a progressive mitochondrial membrane potential (MMP) dissipation starting from 30 min post-treatment without affecting cell viability (Fig. 1C and Suppl. Figs. 1 and 2). Notably, the MMP dissipation observed in HCT116 stimulated with rafoxanide for 1 h was fully reversible upon compound removal, thereby excluding non-specific toxic effects of the drug (Suppl. Fig. 3). Comparable alterations in MMP were detected in DLD1 cells (Suppl. Fig. 4A) but not in the human normal colonic epithelial cell line HCEC-1CT (Suppl. Fig. 5A).

### Prolonged treatment with rafoxanide affects mitochondrial function and promotes cytochrome *c* release to the cytosol

**Fig. 2:**
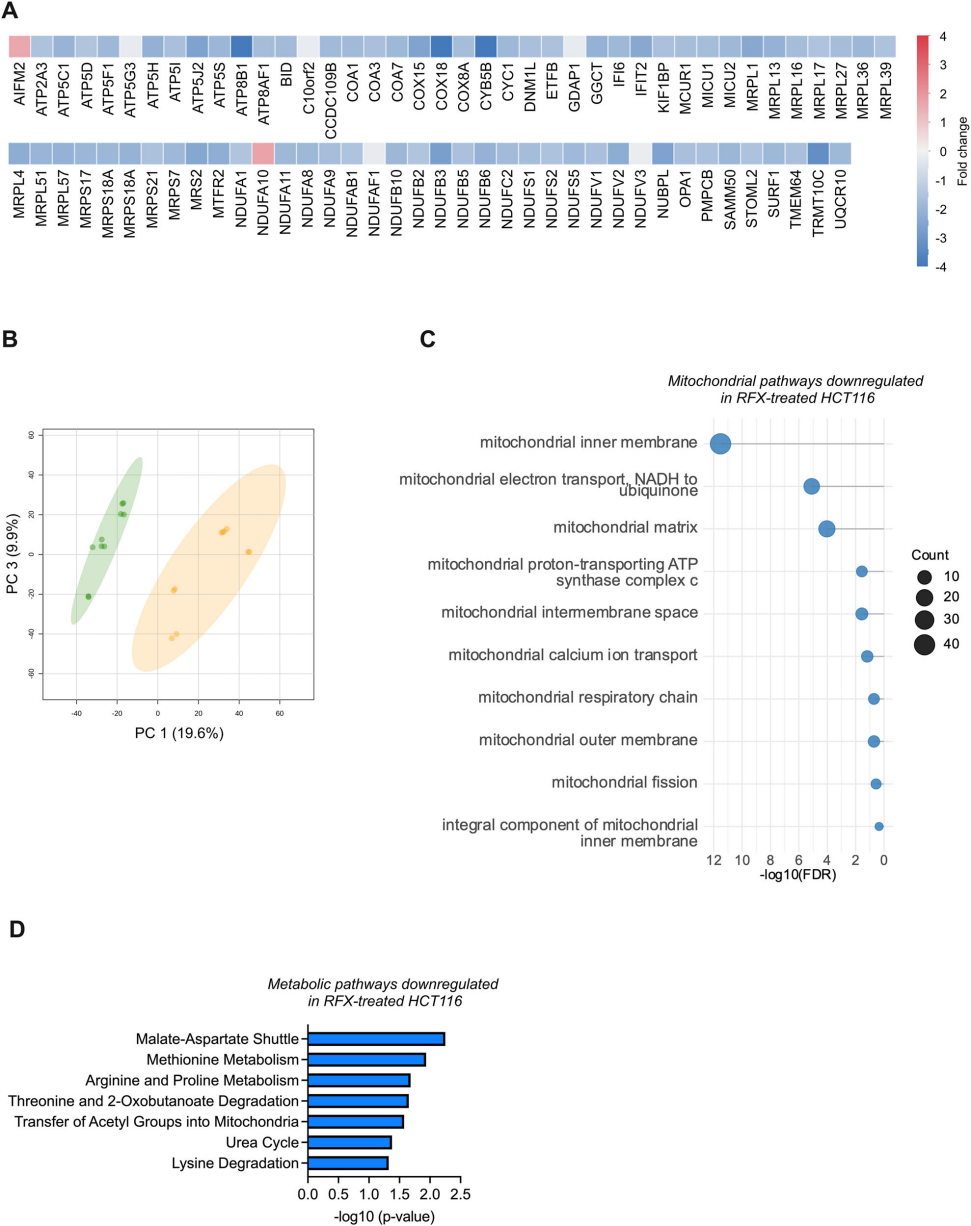
Prolonged treatment with rafoxanide affects mitochondrial function. **A** Heatmap showing the differentially abundant genes associated with mitochondria, ranked by log2 fold-change (log2FC). Results were obtained from the comparison between HCT116 cells treated with rafoxanide (RFX, 2.5 μM) or DMSO (vehicle) for 24 h. **B** Principal Component Analysis (PCA) representation of the proteomic profile of HCT116 cells treated with rafoxanide (orange) or DMSO (green) for 24 h. **C** Lollipop plot showing the top 10 downregulated proteomic pathways ranked by -log10 false discovery rate (-log10FDR), calculated on the set of significantly downregulated mitochondrial proteins in HCT116 cells treated as indicated in panel (**B**). **D** Metabolic pathway enrichment analysis performed using SMPDB (The Small Molecule Pathway Database) on the set of significantly downregulated metabolites identified in HCT116 cells treated as indicated in panel (**B**).

**Fig. 3:**
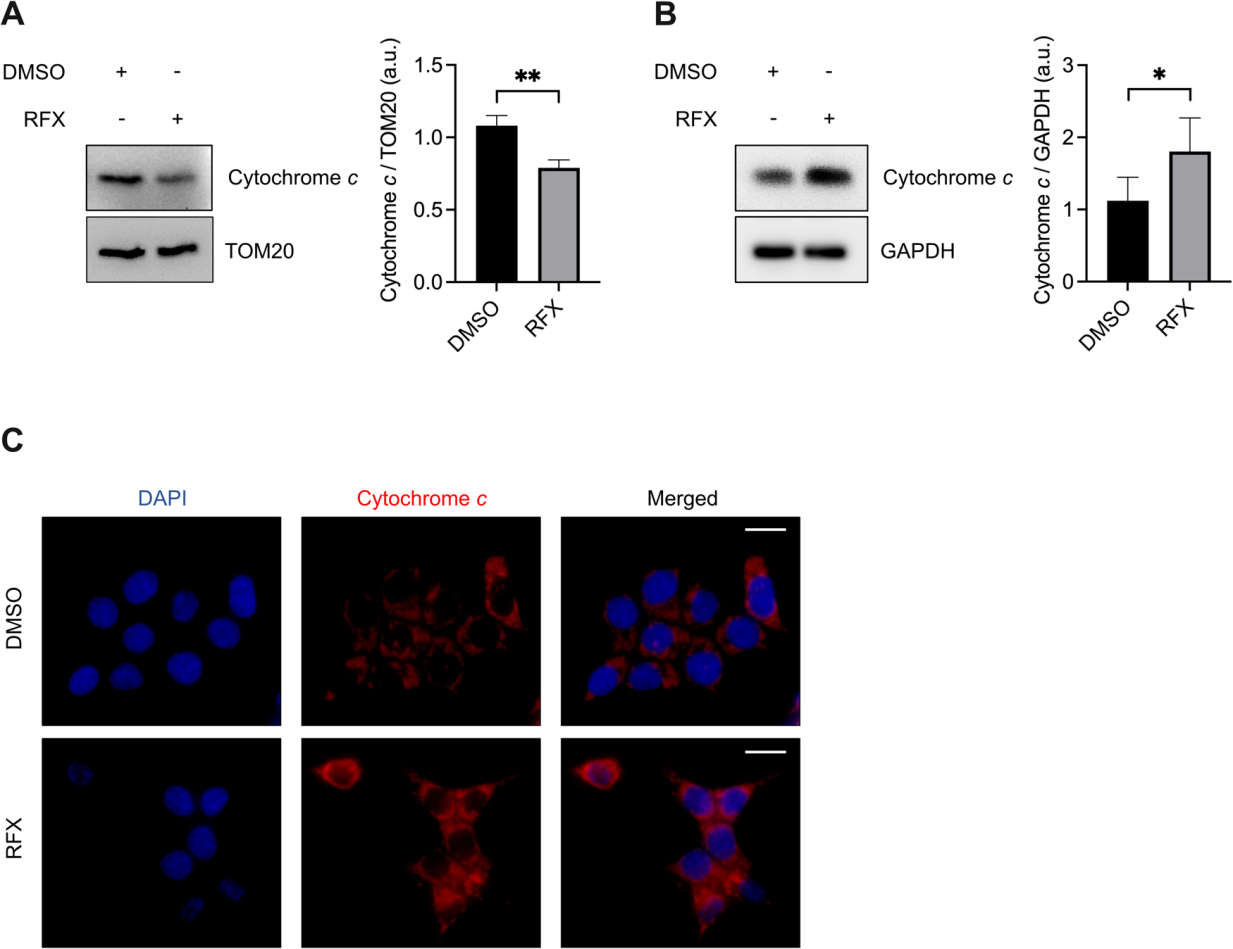
Prolonged rafoxanide treatment promotes cytochrome *c* release into the cytosol. **A** Representative western blot and densitometry analysis of cytochrome *c* and TOM20 positive bands in mitochondrial fractions from HCT116 cells treated with rafoxanide (RFX, 2.5 μM) or DMSO (vehicle) for 24 h. Values are mean ± SEM of 3 independent experiments. Differences between groups were analyzed using a two-tailed Student’s *t*-test (**P ≤ 0.01). **B** Representative western blots and densitometry analysis of cytochrome *c* and GAPDH positive bands in cytosolic fractions from HCT116 cells treated as indicated in panel (**A**). Values are mean ± SEM of 3 independent experiments. Differences between groups were analyzed using a two-tailed Student’s *t*-test (*P ≤ 0.05). **C** Representative immunofluorescence images of the localization of cytochrome *c* (red) within HCT116 cells treated as indicated in panel (**A**). In control cells, cytochrome *c* displays a punctate mitochondrial staining pattern, whereas rafoxanide treatment results in a diffuse cytosolic distribution. Nuclei are stained with DAPI (blue).

To evaluate the effects of prolonged rafoxanide treatment on CRC cell mitochondrial function, we analyzed the transcriptomic, proteomic, and metabolomic profiles of HCT116 and DLD1 cells treated with the drug for 24 h, a time point selected based on previous observations that rafoxanide exerts antitumor activity without inducing overt cell death at this stage [16]. Data from the transcriptome analysis revealed a significant downregulation of several genes related to mitochondrial components and activity in rafoxanide-treated CRC cells compared to the control (DMSO) (120 out of 137 genes in HCT116 cells and 245 out of 370 genes in DLD1 cells) (Fig. 2A and Suppl. Fig. 4B). In particular, the most downregulated genes in HCT116 cells include *ATP8B1*, that regulates mitochondrial membrane lipid composition, *COX18*, which is critical for Complex IV assembly and oxidative phosphorylation, and *CYB5B*, which supports mitochondrial lipid/steroid metabolism via electron transfer (Fig. 2A). In DLD1 cells, instead, ATP synthase assembly resulted to be compromised, with *ATP5G3* and *ATPJ2* genes among the most downregulated genes, together with *TRM10CT* gene, which is involved in mitochondrial tRNA processing/modification (Suppl. Fig. 4B). Interestingly, these alterations were not observed in HCEC-1CT cells (Suppl. Fig. 5B). Proteomic analysis confirmed a decreased expression of proteins associated with mitochondrial components and function in rafoxanide-treated HCT116 cells compared to control (DMSO). In particular, most of them were associated with the mitochondrial inner membrane (e.g., several mitochondrial ribosomal small (MRPS) and large (MRPL) subunit proteins, and electron transfer within the mitochondrial respiratory chain, in particular mitochondrial Complex I (e.g., NDUFA1, NDUFA9, NDUFS) (Fig. 2B, C). Similar results were obtained in rafoxanide-treated DLD1 cells (Suppl. Fig. 4C, D). Additionally, both cell lines exhibited significant downregulation of common metabolic pathways, including the malate-aspartate shuttle, urea cycle, methionine metabolism, and arginine and proline metabolism, confirming mitochondrial dysfunction, energy imbalance, and oxidative stress. (Fig. 2D and Suppl. Fig. 4E). These transcriptional and proteomic changes suggest that CRC cells fail to adapt to sustained mitochondrial stress induced by rafoxanide. In line with this hypothesis, prolonged rafoxanide exposure further exacerbated MMP depolarization at 12 and 24 h, which was accompanied by the translocation of cytochrome *c* from the mitochondria to the cytosol (Fig. 3 and Suppl. Fig. 6), a hallmark of early apoptotic signaling [18, 19]. At later time points (36 h), these mitochondrial alterations were followed by increased cell death (Suppl. Fig. 6). Collectively, these findings demonstrate that rafoxanide induces persistent mitochondrial impairment in CRC cells, ultimately committing them to apoptotic cell death.

### Rafoxanide promotes VDAC1 opening in CRC cells

**Fig. 4:**
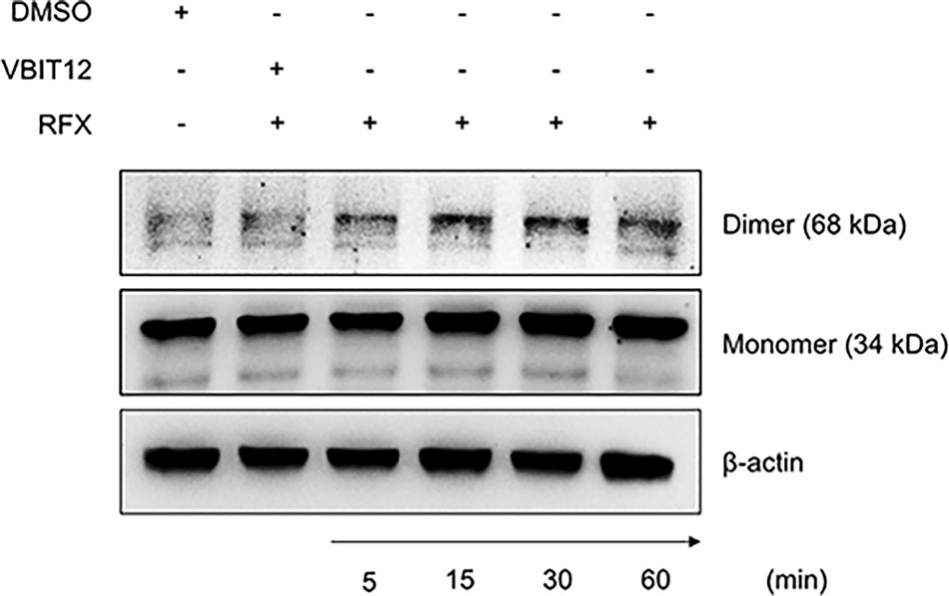
Rafoxanide promotes VDAC1 oligomerization. Representative western blots showing VDAC1 monomeric and dimeric forms, together with β-actin, in HCT116 cells pre-treated or not with the specific VDAC1 inhibitor VBIT12 (40 µM) for 2 h and then stimulated with rafoxanide (RFX, 2.5 μM) or DMSO (vehicle) for 5, 15, 30, and 60 min. One of 3 independent experiments in which similar results were obtained is shown.

A key step in mitochondria-mediated apoptosis is the oligomerization of VDAC1, a highly abundant protein of the mitochondrial outer membrane [2, 20]. Upon stimulation by apoptotic inducers, VDAC1 forms a large channel that allows the release of cytochrome *c* and other pro-apoptotic proteins, as well as metabolites, from mitochondria into the cytosol [3, 8, 21–23]. We therefore investigated whether rafoxanide could induce mitochondrial dysfunction by promoting the opening of VDAC1. HCT116 cells were treated with rafoxanide for different time points (i.e., 5, 15, 30, and 60 min), and the oligomeric state of VDAC1 was assessed by western blotting. Of note, VDAC1 dimer levels were increased as early as 5 min following rafoxanide treatment compared to control (DMSO) (Fig. 4). As expected, pre-treatment with a specific VDAC1 inhibitor (VBIT12, 40 µM) effectively prevented rafoxanide-induced VDAC1 oligomerization at 60 min (Fig. 4). Overall, these results indicate that rafoxanide promotes VDAC1 oligomerization, thereby facilitating cytochrome *c* release from mitochondria to the cytosol.

### Rafoxanide promotes VDAC1 opening through mitochondrial respiratory chain impairment and induction of cellular oxidative stress

**Fig. 5:**
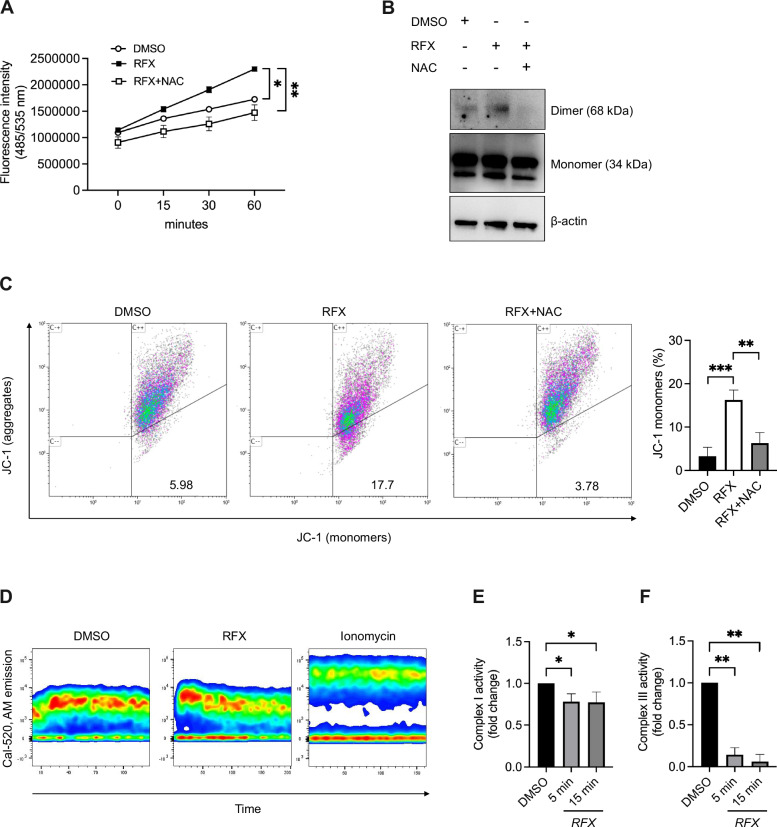
Rafoxanide-induced inhibition of mitochondrial complexes I and III triggers a ROS-dependent VDAC1 activation and mitochondrial membrane depolarization in CRC cells. **A** Detection of total cellular reactive oxygen species (ROS) by fluorescence intensity measurement in HCT116 cells pre-treated or not with the ROS scavenger NAC (1 mM) for 1 h and then incubated with fluorescence probe DCFDA. After 30 min, cells were washed and stimulated with rafoxanide (RFX, 2.5 μM) or DMSO (vehicle). Fluorescence intensity was measured at the baseline and after 5, 15, 30, and 60 min upon stimulation. Differences among groups were compared using one-way analysis of variance (ANOVA) followed by the Tukey’s post hoc test (*P ≤ 0.05, **P ≤ 0.01). One of 3 independent experiments where similar results were obtained. **B** Representative western blots showing VDAC1 monomeric and dimeric forms, together with β-actin, in HCT116 cells pre-treated or not with the ROS scavenger NAC (1 mM) for 1 h and then stimulated with rafoxanide (RFX, 2.5 μM) or DMSO (vehicle) for 1 h. One of 3 independent experiments in which similar results were obtained is shown. **C** *Left panel*: representative flow cytometry dot plots showing JC-1 fluorescence in HCT116 cells treated as indicated in panel (**B**). JC-1 aggregates indicate polarized mitochondria, whereas JC-1 monomers indicate mitochondrial membrane depolarization. Numbers indicate the percentage of cells in the designated quadrants. *Right panel*: quantification of the fraction of cells with depolarized mitochondria. Values are mean ± SEM of 3 independent experiments. Differences among groups were compared using one-way ANOVA followed by Tukey’s post hoc test (**P ≤ 0.01, ***P ≤ 0.001). **D**, **E** Complex I (**D**) and complex III (**E**) activity assays were performed spectrophotometrically in HCT116 cells stimulated with rafoxanide (RFX, 2.5 μM) or DMSO (vehicle) for 5 and 15 min. Values are mean ± SEM of 3 independent experiments. Differences among groups were compared using one-way ANOVA followed by Tukey’s post hoc test (*P ≤ 0.05, **P ≤ 0.01). **F** Calcium mobilization was measured by flow cytometry in HCT116 cells stained with the 520-AM dye for 30 min, washed, and stimulated with rafoxanide (RFX, 2.5 μM), DMSO (vehicle), or ionomycin (positive control). Samples were acquired by flow cytometry at the baseline and immediately after stimulation. One of 3 independent experiments in which similar results were obtained is shown.

We next sought to elucidate the mechanism underlying rafoxanide-mediated VDAC1 opening. Although the exact molecular events governing VDAC1 oligomerization remain incompletely defined, previous studies suggest that this process is modulated by intracellular Ca^2+^ levels and redox status [24, 25]. We first assessed ROS production following rafoxanide treatment. A rapid increase in total cellular ROS was detected in HCT116 cells as early as 5 min after treatment with rafoxanide compared to control (DMSO) (Fig. 5A). Pre-treatment with N-acetyl-l-cysteine (NAC, 1 mM), a potent ROS scavenger, markedly reduced VDAC1 oligomerization and attenuated mitochondrial membrane potential dissipation induced by rafoxanide at 60 min (Fig. 5B, C), indicating that ROS signaling plays a key role in promoting VDAC1 opening and mitochondrial dysfunction. In contrast, stimulation with rafoxanide did not elicit a robust increase in cytosolic Ca^2+^ levels under our experimental conditions (Fig. 5D), suggesting that Ca²⁺ flux modulation is not the primary driver of VDAC1 activation in this context. Since impairment of OXPHOS is known to disrupt electron flow within the mitochondrial respiratory chain, leading to altered redox homeostasis [26], we investigated the effect of rafoxanide on respiratory chain activity. Exposure to rafoxanide for 5 or 15 min significantly inhibited the activity of both mitochondrial complex I and complex III in HCT116 cells (Fig. 5E, F). Despite this early inhibition, mitochondrial superoxide production measured by MitoSOX was reduced following rafoxanide treatment, starting from 15 min (Suppl. Fig. 7). This apparent discrepancy is consistent with the progressive loss of mitochondrial membrane potential observed upon rafoxanide exposure, as mitochondrial depolarization is known to suppress superoxide generation at the respiratory chain despite the presence of a broader cellular oxidative stress response [27].

Collectively, these findings indicate that rafoxanide rapidly inhibits mitochondrial complexes I and III, leading to mitochondrial depolarization and suppression of mitochondrial superoxide production, while concomitantly inducing a ROS-dependent cellular stress response that promotes VDAC1 opening and contributes to mitochondrial dysfunction in CRC cells.

### Rafoxanide induces mitochondrial dysfunction in murine and patient-derived CRC models

**Fig. 6:**
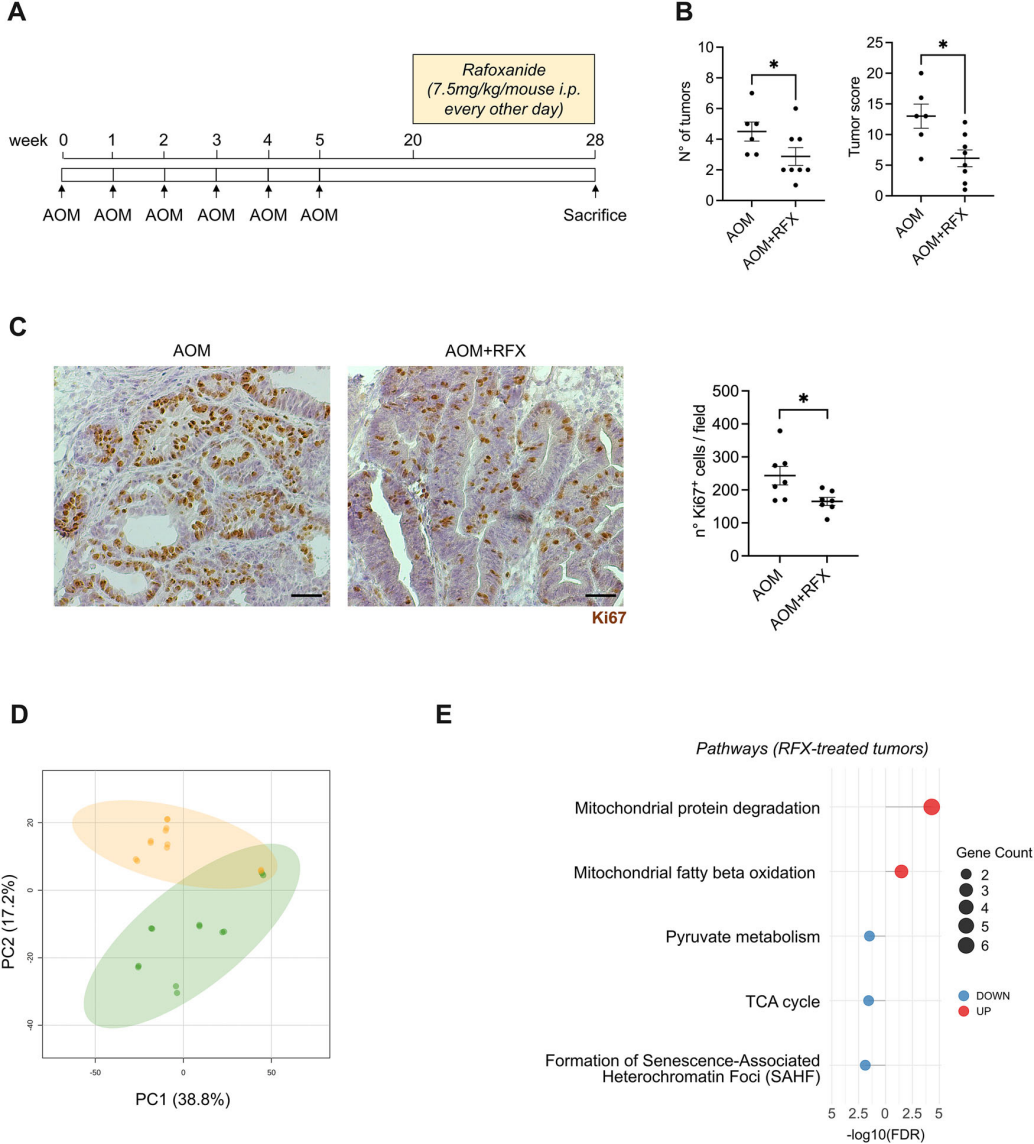
Rafoxanide alters mitochondrial activity in vivo. **A** Schematic overview of the experimental protocol used to induce sporadic CRC in mice. **B** Scatter plots showing the number and size of tumors developed in mice receiving an intraperitoneal (i.p.) injection of azoxymethane (AOM; 10 mg/kg) once weekly for 6 weeks. After 20 weeks following the first injection, mice were treated with rafoxanide (AOM + RFX, 7.5 mg/Kg, i.p, *n* = 8) or not (AOM, *n* = 6) every other day, for an additional 8 weeks. Values are mean ± SEM, and each point represents a single mouse. Differences between groups were analyzed using a two-tailed Student’s *t*-test (***P ≤ 0.001). **C** Representative immunohistochemistry images and quantification of Ki67-positive cells in colonic tumors from mice treated as indicated in panel (**B**). Scale bars: 50 μm. The scatter plot indicates the mean number of Ki67-positive cells per field, counted in four different fields per section. Differences were analyzed using a two-tailed Student’s *t*-test (*P ≤ .05). **D** Principal Component Analysis (PCA) representation of the proteomic profile of colonic tumors isolated from mice treated (orange) or not (green) with rafoxanide, as indicated in panel (**B**). **E** Lollipop plot showing the downregulated (blue) and upregulated (red) proteomic pathways ranked by -log10 false discovery rate (-log10FDR), calculated on the set of significantly downregulated and upregulated mitochondrial proteins in colonic tumors isolated from mice treated as indicated in panel (**B**).

**Fig. 7:**
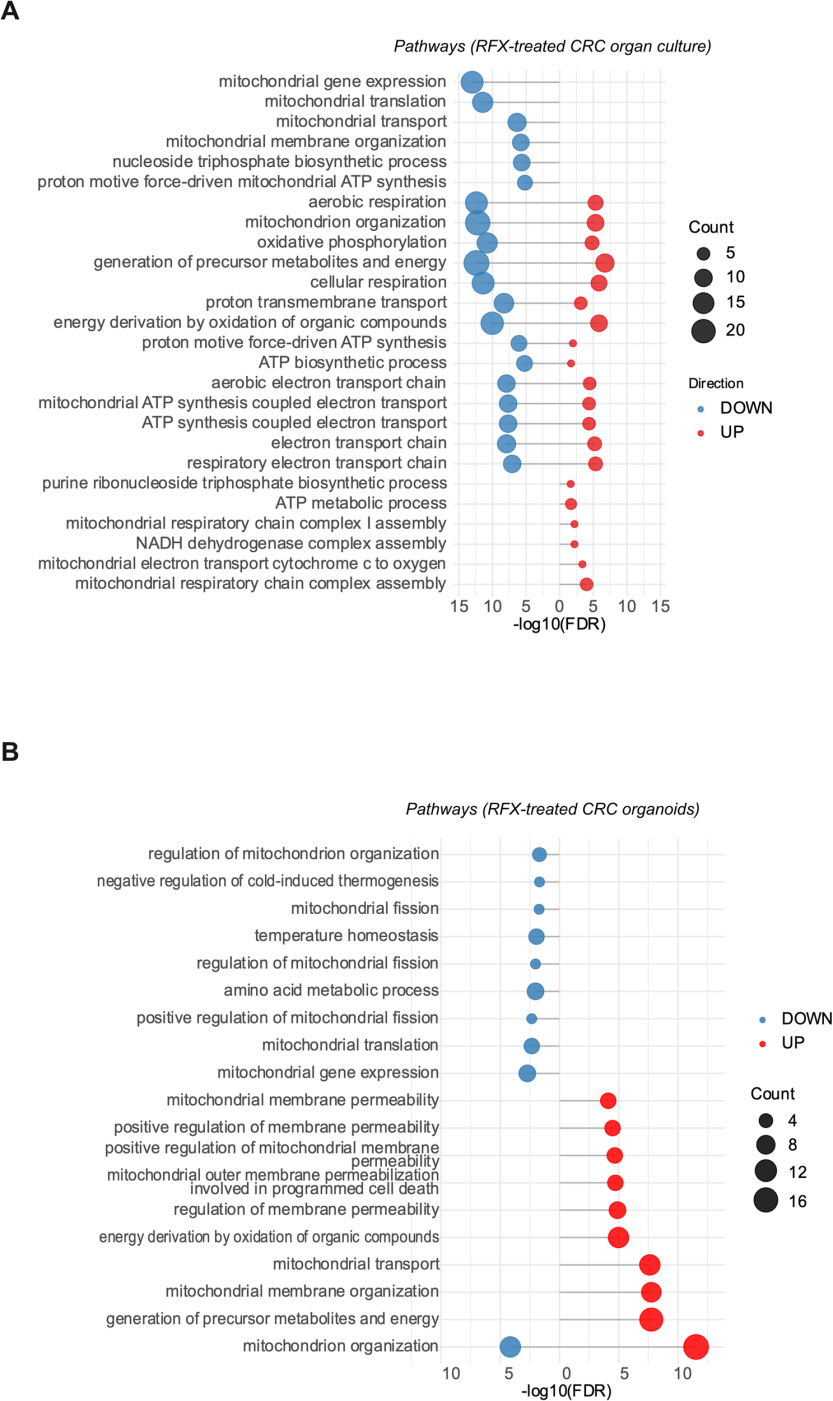
Rafoxanide alters mitochondrial activity in CRC patient-derived samples. **A** Lollipop plot showing the downregulated (blue) and upregulated (red) proteomic pathways ranked by -log10 false discovery rate (−log10FDR), calculated on the set of significantly downregulated and upregulated mitochondrial proteins from human CRC explants treated (*n* = 6) or not (*n* = 6) with rafoxanide (10 μM) for 16 h. **B** Lollipop plot showing the downregulated (blue) and upregulated (red) proteomic pathways ranked by -log10 false discovery rate (-log10FDR), calculated from the set of differentially expressed mitochondrial proteins in CRC patient-derived intestinal organoids treated with rafoxanide (10 μM, *n* = 3) or vehicle (DMSO, *n* = 3) for 24 h.

Subsequently, we evaluated whether rafoxanide affected mitochondrial function in vivo using a murine model mimicking sporadic CRC (Fig. 6A). At week 28, animals were sacrificed and colonic lesions collected for proteomic analysis. Rafoxanide-treated mice developed fewer and smaller lesions compared to controls (Fig. 6B), with a reduced frequency of Ki67^+^ proliferative tumor cells (Fig. 6C), indicating an inhibitory effect on tumor growth in vivo. Proteomic profiling of colonic tumor samples revealed marked alteration in mitochondrial protein expression following rafoxanide treatment (Fig. 6D, E). In particular, differential analyses identified 58 mitochondrial proteins that were significantly modulated (41 upregulated and 17 downregulated) in tumors from rafoxanide-treated mice compared with controls. Of note, rafoxanide treatment was associated with downregulation of proteins involved in pyruvate metabolism (e.g., Dld, Fahd1), tricarboxylic acid (TCA) cycle activity (e.g., Dld, Sdhb), and chromatin-associated factors linked to senescence-associated heterochromatin organization (e.g., Hmga1, Hmga1b), which are known to be influenced by mitochondrial dysfunction [28] (Fig. 6E). In contrast, proteins involved in mitochondrial protein degradation (such as Clpp) and fatty acid beta-oxidation (e.g., Acadvl, Acot2, Hadha) were upregulated, suggesting a state of metabolic stress and a compensatory shift in metabolism due to the need for alternative energy sources (Fig. 6E). In line with these findings, mitochondrial proteomic analysis performed on CRC patient-derived explants and intestinal organoids treated with rafoxanide also revealed marked changes in mitochondrial protein expression (Fig. 7A, B). Differential analyses in rafoxanide-treated CRC patient-derived explants revealed 136 mitochondrial proteins with altered expression (33 upregulated and 103 downregulated). Most of the proteins that are downregulated were linked to mitochondrial organization, such as several mitochondria ribosomal large subunit proteins (e.g., Mrpl-11, -15, -27, -45, -49, and -51), which are essential for protein synthesis within mitochondria, as well as proteins involved in electron transport chain activity (e.g., Atp5f1e, Atp5pf, Cox5b, Cox6c, Cox7a2l, Ndufa7, Ndufa8, and Ndufs6) (Fig. 7A). Mitochondrial proteomic profiling in human CRC organoids (a total of 99 differentially expressed mitochondrial proteins, with 55 upregulated and 44 downregulated) confirmed a significant impairment of proteins related to mitochondrial gene expression (such as mitochondrial ribosomal subunit proteins like Mrps9 and Mrps30, as well as Mrpl20 and Mrpl49) and fission (including Pgam5, Mff, Marchf5) following rafoxanide treatment (Fig. 7B). Importantly, several proteins associated with mitochondrial membrane permeabilization and stress signaling were found to be modulated in rafoxanide-treated samples (e.g., Bcl2l1, Ppif), consistent with enhanced mitochondrial membrane permeability and dysfunction (Fig. 7B).

Altogether, these in vivo and ex vivo data support the notion that rafoxanide induces mitochondrial stress and dysfunction in CRC tissues, in agreement with our in vitro findings.

## Discussion

In this study, we investigated the impact of the halogenated salicylanilide rafoxanide on mitochondrial homeostasis in CRC cells. Our results demonstrate that rafoxanide induces a progressive and selective mitochondrial dysfunction, instead of a sudden bioenergetic collapse, affecting CRC cells both in vitro and in vivo while sparing normal colonic epithelial cells. This mitochondrial stress response is characterized by early inhibition of mitochondrial respiration, gradual dissipation of MMP, and failure of cancer cells to adapt to sustained mitochondrial perturbation. Rafoxanide rapidly impaired mitochondrial oxygen consumption and reduced both basal and maximal respiration, indicating an early functional inhibition of oxidative phosphorylation. Importantly, this effect was initially reversible, as removal of the drug restored MMP, arguing against non-specific cytotoxicity.

However, prolonged exposure resulted in coordinated transcriptional, proteomic, and metabolic reprogramming that ultimately prevented CRC cells from restoring mitochondrial function. These findings indicate that rafoxanide induces a sustained mitochondrial stress state that CRC cells are unable to compensate for over time.

Mechanistically, our data reveal that rafoxanide inhibits mitochondrial respiratory chain complexes I and III, leading to profound alterations in cellular redox homeostasis. Although mitochondrial superoxide production was reduced by rafoxanide, total cellular ROS levels were rapidly increased. This distinction underlines that rafoxanide elicits a ROS-dependent cellular stress response that contributes to mitochondrial dysfunction rather than acting as a classical pro-oxidant at the mitochondrial level.

A central finding of this work is the identification of VDAC1 as a critical effector linking redox stress to mitochondrial outer membrane permeabilization. VDAC1 oligomerization is a well-established mechanism controlling the release of cytochrome *c* and other pro-apoptotic factors from mitochondria [25]. We demonstrate that rafoxanide promotes rapid and sustained VDAC1 oligomerization in CRC cells, an effect that is significantly attenuated by ROS scavenging. These observations support a model in which redox imbalance induced by mitochondrial respiratory chain inhibition promotes VDAC1 opening, thereby committing cells to apoptosis [20, 29]. While we cannot exclude the possibility that rafoxanide may also directly interact with VDAC1, this remains speculative and will require future structural and biochemical studies.

A striking feature of rafoxanide is its selectivity toward CRC cells, sparing normal colonic epithelial cells. Cancer cells typically exhibit elevated basal ROS levels and increased reliance on mitochondrial metabolism, rendering them more susceptible to additional mitochondrial and redox stress. In this context, rafoxanide-induced disruption of mitochondrial function may selectively exceed the adaptive capacity of CRC cells, while normal cells remain largely unaffected.

Our findings also integrate well with our previous observations that rafoxanide induces ER stress and suppresses pro-inflammatory signaling in CRC. While mitochondrial dysfunction was not evident at early time points in our earlier studies (that is, 5–15 min) [16], we now demonstrate that mitochondrial depolarization becomes detectable within 30 min of treatment. We propose that rafoxanide-induced redox imbalance serves as a unifying upstream signal linking mitochondrial dysfunction and ER stress, consistent with literature linking oxidative stress, misfolded protein accumulation, and activation of the unfolded protein response [30, 31].

Importantly, the relevance of these mechanisms extends beyond in vitro systems. By employing a murine model mimicking sporadic CRC, as well as patient-derived CRC explants and intestinal organoids, we demonstrate that rafoxanide alters mitochondrial protein expression and metabolic pathways in tumor tissues. These data reinforce the translational relevance of our findings and indicate that rafoxanide-induced mitochondrial stress is a conserved feature across experimental systems.

Mitochondrial-targeting strategies, including partial uncoupling and modulation of mitochondrial stress responses, have emerged as promising approaches in cancer therapy [32–35]. Previous studies on compounds such as BAM15, nitazoxanide, and niclosamide support the notion that controlled mitochondrial dysfunction can impair tumor growth without inducing overt toxicity. In this context, rafoxanide appears to act as a modulator of mitochondrial homeostasis, rather than as a classical uncoupler, engaging mitochondrial and ER stress pathways to promote CRC cell death.

While our results support the potential of rafoxanide as an anticancer agent, further pharmacological and toxicological evaluation will be required before clinical translation. Notably, rafoxanide is not currently approved for human use, although other halogenated salicylanilides, such as niclosamide, have demonstrated a favorable safety profile and are under clinical investigation in oncology (NCT05188170, NCT02807805) [35, 36]. Our observations in patient-derived organoids, together with preliminary evidence of limited toxicity in non-tumor human cells, provide a rationale for continued preclinical development.

In conclusion, our study identifies rafoxanide as a compound that disrupts mitochondrial homeostasis in CRC cells through respiratory chain inhibition, redox-dependent VDAC1 activation, and progressive mitochondrial dysfunction, ultimately leading to programmed cell death. These findings provide mechanistic insight into the antitumor activity of rafoxanide and support further exploration of mitochondrial stress modulation as a therapeutic strategy in CRC.

## Materials and methods

### Animals

Balb/c mice were obtained from the Charles River Laboratories (Lodi, Italy) and maintained in filter-topped cages on autoclaved food and water at the University of Rome “Tor Vergata” animal facility (Rome, Italy). All animal studies were reviewed and approved by the Institutional Animal Care and Use Committee of the University of Rome Tor Vergata and the Italian Ministry of Health (Protocol 152/2023-PR).

### Experimental model of mouse sporadic CRC

To induce sporadic CRC, co-housed 7–8-week-old female Balb/c mice received an intraperitoneal (i.p.) injection of azoxymethane (AOM, 10 mg/kg, #A5486, Sigma Aldrich, St Louis, Missouri, USA) once a week for 6 weeks [37]. After 20 weeks, mice were randomly divided into two groups and given rafoxanide (7.5 mg/Kg, #R0108, Tokyo Chemical Industry Co., Ltd., Tokyo, Japan) or vehicle [10% DMSO (#D2438, Sigma Aldrich) in PBS], every other day by i.p. injection until sacrifice (week 28). Colonoscopy was carried out two days before sacrifice in a blinded manner for monitoring of tumorigenesis using the Coloview high-resolution mouse endoscopic system (Karl Storz, Tuttlingen, Germany). The lesions observed during endoscopy were counted to obtain the total number of lesions. The size of all lesions in a given mouse was scored using the protocol described by Becker et al. [38]. The tumor score of each lesion was less than the maximum approved by the local Institutional Animal Care and Use Committee.

The dose of rafoxanide was selected according to the one currently used in veterinary treatment (i.e., 7.5–10 mg/kg).

### Patients

Tissue samples were taken from the tumor area of 9 patients who underwent colon resection for sporadic CRC (all with TNM stages II–III) at the Tor Vergata University Hospital and used for intestinal organ culture or to generate intestinal organoids. No patients received radiation therapy or chemotherapy prior to surgery. All human research was reviewed and approved by the ethics committee of the Tor Vergata University Hospital (protocol n° R.S.131.17). Informed consent was obtained from all human research participants. The study methodologies adhered to the standards set by the Declaration of Helsinki.

### Cell cultures and reagents

The human CRC cell line HCT-116 was obtained from ATCC and maintained in McCoy’s 5 A medium (#MCC-A, Capricorn Scientific, Ebsdorfergrund, Germany) supplemented with 10% fetal bovine serum (FBS) (D104#, Diagnovum, Greifswald, Germany) and 1% penicillin/streptomycin (P/S, #PS-B, Capricorn Scientific). The human CRC cell line DLD1 was obtained from ATCC and was maintained in RPMI-1640 medium (#RPMI-STA, Capricon Scientific) supplemented with 10% FBS and 1% P/S. The human normal colonic epithelial cell line HCEC-1CT was obtained from EVERCYTE GmbH (Vienna, Austria) and maintained in ColoUp medium (#MHT-039, EVERCYTE GmbH).

Cell lines were recently authenticated by short tandem repeat (STR) DNA fingerprinting using the PowerPlex 18D System kit according to the manufacturer’s instructions (#DC1802, Promega, Madison, Wisconsin, USA). The STR profiles of all cell lines matched the known DNA fingerprints. HCT116 cells were pre-treated for 2 h with the VDAC1 inhibitor VBIT12 (40 µM, #S8936, Selleck Chemicals LLC, Houston, Texas) to block VDAC1 oligomerization or for 1 h with N-acetylcysteine (NAC, 1 mM, #A0150000, Sigma Aldrich) to decrease reactive oxygen species (ROS) level.

### Organ culture

Organ culture experiments were carried out as previously described [39]. Briefly, human CRC explants were placed on Millicell inserts in a 6-well plate containing complete RPMI-1640 medium in the presence of DMSO (vehicle) or 10 μM rafoxanide for 16 h. Culture was carried out in an organ culture chamber at 37 °C in a 5% CO_2_/95% O_2_ atmosphere.

### Patient-derived organoids

Patient-derived organoids were established and maintained in culture medium as previously described [40], and expanded by passaging every 5–7 days. For proteomic analyses, 1 × 10^6^ cells to form organoids per condition were seeded in 6-well plates within 5 domes of Matrigel® (#CLS356239, Corning, New York, USA) and overlaid with complete growth medium. After overnight culture, organoids were treated with rafoxanide (10 μM) or vehicle (DMSO). Samples were harvested at 24 h post-treatment and pelleted for further analyses.

### Chemical cross-linking

HCT116 and DLD1 were treated with rafoxanide (2.5 μM) or vehicle (DMSO) for 15, 30, and 60 min. Cells were then washed twice with PBS and cross-linked by incubation with ethylene glycol bis (succinimidyl succinate) (EGS, 0.5 mM, pH 7.4) for 30 min at room temperature. The reaction was quenched by adding Tris-HCl 1.5 M, pH 7.8, for 5 min. Cells were then centrifuged at 12000 g for 5 min and lysed on ice. Samples were subjected to SDS-PAGE and immunoblotting using an anti-VDAC1 antibody (1:500 dilution, sc-390996, Santa Cruz Biotechnology, Dallas, Texas, USA)

### Flow cytometry measurement of mitochondrial membrane potential

To measure the mitochondrial membrane potential, HCT116, DLD1, and HCEC-1CT cells were stimulated with rafoxanide (2.5 μM) or vehicle (DMSO) for 15, 30, and 60 min. Cells were harvested and stained with the tetraethylbenzimidazolylcarbocyanine iodide dye (JC1, 4 µM, #T3168, Invitrogen) for 15 min or with 3,3’-Dihexyloxacarbocyanine Iodide (DiOC6(3), 40 nM, #D273, Invitrogen) dye and propidium iodide (PI, 5 μg/ml, # 537060, Sigma Aldrich) for 20 min at 37 °C. Cells were then washed with PBS and analyzed by flow cytometry.

### Reactive oxygen species detection

Intracellular ROS production was assessed in HCT116 cells using the 2′,7′–dichlorofluorescin diacetate (DCFDA) Cellular ROS Detection Assay Kit (#ab113851, Abcam, Cambridge, United Kingdom) according to the manufacturer’s protocol and measured using DXT880 Multimode detector (Excitation 488 nm, Emission: 530 nm, Beckman Coulter, Brea, California, USA). Levels of mitochondrial ROS were quantified by flow cytometry using the mitochondrial superoxide indicator MitoSOX (#M36008, Invitrogen) according to the manufacturer’s instructions.

### Intracellular Ca2^+^ measurement

Intracellular Ca2^+^ mobilization was assessed by flow cytometry (Excitation 488 nm, Emission: 530 nm) using the fluorescence-based assay Calcium Flux Assay Kit (Cal-520 AM dye, #ab233472, Abcam) according to the manufacturer’s protocol.

### Statistical analysis

Parametric data were analyzed using the two-tailed Student’s *t*-test for comparison between two groups or one-way analysis of variance (ANOVA) followed by Tukey’s post hoc test for multiple comparisons. Significance was defined as *p* < 0.05.

## Supplementary information

Suppl. Figure 1

Suppl. Figure 2

Suppl. Figure 3

Suppl. Figure 4

Suppl. Figure 5

Suppl. Figure 6

Suppl. Figure 7

Suppl. figure legend

Suppl. materials&methods

uncropped wb

## Data Availability

The data generated during this study are available from the corresponding author upon reasonable request.

## References

[1] McBride HM, Neuspiel M, Wasiak S. Mitochondria: more than just a powerhouse. Curr Biol. 2006;16:R551–60. 16860735 10.1016/j.cub.2006.06.054

[2] Shoshan-Barmatz V, De S, Meir A. The mitochondrial voltage-dependent anion channel 1, Ca2+ transport, apoptosis, and their regulation. Front Oncol 2017;7. 10.3389/fonc.2017.00060. 10.3389/fonc.2017.00060 PMC5385329 28443244

[3] Tomasello F, Messina A, Lartigue L, Schembri L, Medina C, Reina S, et al. Outer membrane VDAC1 controls permeability transition of the inner mitochondrial membrane in cellulo during stress-induced apoptosis. Cell Res. 2009;19:1363–76. 19668262 10.1038/cr.2009.98

[4] Rostovtseva TK, Sheldon KL, Hassanzadeh E, Monge C, Saks V, Bezrukov SM, et al. Tubulin binding blocks mitochondrial voltage-dependent anion channel and regulates respiration. Proc Natl Acad Sci USA. 2008;105:18746–51. 19033201 10.1073/pnas.0806303105 PMC2596221

[5] Abu-Hamad S, Zaid H, Israelson A, Nahon E, Shoshan-Barmatz V. Hexokinase-I protection against apoptotic cell death is mediated via interaction with the voltage-dependent anion channel-1. J Biol Chem. 2008;283:13482–90. 18308720 10.1074/jbc.M708216200

[6] Keinan N, Tyomkin D, Shoshan-Barmatz V. Oligomerization of the mitochondrial protein voltage-dependent anion channel is coupled to the induction of apoptosis. Mol Cell Biol. 2010;30:5698–709. 20937774 10.1128/MCB.00165-10 PMC3004265

[7] Smith ALM, Whitehall JC, Greaves LC. Mitochondrial DNA mutations in ageing and cancer. Mol Oncol. 2022;16:3276–94. 35842901 10.1002/1878-0261.13291 PMC9490137

[8] Tsujimoto Y, Shimizu S. Role of the mitochondrial membrane permeability transition in cell death. Apoptosis. 2007;12:835–40. 17136322 10.1007/s10495-006-0525-7

[9] Uslu C, Kapan E, Lyakhovich A. Cancer resistance and metastasis are maintained through oxidative phosphorylation. Cancer Lett. 2024;587:216705. 38373691 10.1016/j.canlet.2024.216705

[10] Isono T, Chano T, Yonese J, Yuasa T. Therapeutic inhibition of mitochondrial function induces cell death in starvation-resistant renal cell carcinomas. Sci Rep. 2016;6:25669. 27157976 10.1038/srep25669 PMC4860706

[11] Vasan K, Werner M, Chandel NS. Mitochondrial metabolism as a target for cancer therapy. Cell Metab. 2020;32:341–52. 32668195 10.1016/j.cmet.2020.06.019 PMC7483781

[12] Demine S, Renard P, Arnould T. Mitochondrial uncoupling: a key controller of biological processes in physiology and diseases. Cells. 2019;8:795. 31366145 10.3390/cells8080795 PMC6721602

[13] Shrestha R, Johnson E, Byrne FL. Exploring the therapeutic potential of mitochondrial uncouplers in cancer. Mol Metab. 2021;51:101222. 33781939 10.1016/j.molmet.2021.101222 PMC8129951

[14] Weinbach EC, Garbus J. Mechanism of action of reagents that uncouple oxidative phosphorylation. Nature. 1969;221:1016–8. 4180173 10.1038/2211016a0

[15] Prichard RK. The metabolic profile of adult *Fasciola hepatica* obtained from rafoxanide-treated sheep. Parasitology. 1978;76:277–88. 26902 10.1017/s0031182000048150

[16] Laudisi F, Di Grazia A, De Simone V, Cherubini F, Colantoni A, Ortenzi A, et al. Induction of endoplasmic reticulum stress and inhibition of colon carcinogenesis by the anti-helmintic drug rafoxanide. Cancer Lett*.* 2019; 462. 10.1016/j.canlet.2019.07.014. 10.1016/j.canlet.2019.07.014 31351087

[17] Pacifico T, Stolfi C, Tomassini L, Luiz-Ferreira A, Franzè E, Ortenzi A, et al. Rafoxanide negatively modulates STAT3 and NF-κB activity and inflammation-associated colon tumorigenesis. Cancer Sci. 2024;115:3596–611. 39239848 10.1111/cas.16317 PMC11531958

[18] Cai J, Yang J, Jones DeanP. Mitochondrial control of apoptosis: the role of cytochrome c. Biochim Biophys Acta Bioenerg. 1998;1366:139–49. 10.1016/s0005-2728(98)00109-1 9714780

[19] Kluck RM, Bossy-Wetzel E, Green DR, Newmeyer DD. The release of cytochrome c from mitochondria: a primary site for Bcl-2 regulation of apoptosis. Science. 1997;275:1132–6. 9027315 10.1126/science.275.5303.1132

[20] Colombini M. VDAC: The channel at the interface between mitochondria and the cytosol. Mol Cell Biochem. 2004;256–257: 107–115. 10.1023/b:mcbi.0000009862.17396.8d 14977174

[21] Zalk R, Israelson A, Garty ES, Azoulay-Zohar H, Shoshan-Barmatz V. Oligomeric states of the voltage-dependent anion channel and cytochrome *c* release from mitochondria. Biochem J. 2005;386:73–83. 15456403 10.1042/BJ20041356 PMC1134768

[22] Shoshan-Barmatz V, Keinan N, Zaid H. Uncovering the role of VDAC in the regulation of cell life and death. J Bioenerg Biomembr. 2008;40:183–91. 18651212 10.1007/s10863-008-9147-9

[23] Shoshan-Barmatz V, De Pinto V, Zweckstetter M, Raviv Z, Keinan N, Arbel N. VDAC, a multi-functional mitochondrial protein regulating cell life and death. Mol Asp Med. 2010;31:227–85. 10.1016/j.mam.2010.03.002 20346371

[24] Gincel D, Zaid H, Shoshan-Barmatz V. Calcium binding and translocation by the voltage-dependent anion channel: a possible regulatory mechanism in mitochondrial function. Biochem J. 2001;358:147. 11485562 10.1042/0264-6021:3580147 PMC1222042

[25] Madesh M, Hajnóczky G. VDAC-dependent permeabilization of the outer mitochondrial membrane by superoxide induces rapid and massive cytochrome *c* release. J Cell Biol. 2001;155:1003–16. 11739410 10.1083/jcb.200105057 PMC2150912

[26] Tirichen H, Yaigoub H, Xu W, Wu C, Li R, Li Y. Mitochondrial reactive oxygen species and their contribution in chronic kidney disease progression through oxidative Stress. Front Physiol. 2021;12:627837. 10.3389/fphys.2021.627837 PMC8103168 33967820

[27] Akude E, Zherebitskaya E, Chowdhury SKR, Smith DR, Dobrowsky RT, Fernyhough P. Diminished superoxide generation is associated with respiratory chain dysfunction and changes in the mitochondrial proteome of sensory neurons from diabetic rats. Diabetes. 2011;60:288–97. 20876714 10.2337/db10-0818 PMC3012184

[28] Correia-Melo C, Marques FD, Anderson R, Hewitt G, Hewitt R, Cole J, et al. Mitochondria are required for pro-ageing features of the senescent phenotype. EMBO J. 2016;35:724–42. 26848154 10.15252/embj.201592862 PMC4818766

[29] Rui H, Lee KIL, Pastor RW, Im W. Molecular dynamics studies of ion permeation in VDAC. Biophys J. 2011;100:602–10. 21281574 10.1016/j.bpj.2010.12.3711 PMC3030152

[30] Malhotra JD, Kaufman RJ. Endoplasmic reticulum stress and oxidative stress: a vicious cycle or a double-edged sword?. Antioxid Redox Signal. 2007;9:2277–94. 17979528 10.1089/ars.2007.1782

[31] Malhotra JD, Miao H, Zhang K, Wolfson A, Pennathur S, Pipe SW, et al. Antioxidants reduce endoplasmic reticulum stress and improve protein secretion. Proc Natl Acad Sci. 2008;105:18525–30. 19011102 10.1073/pnas.0809677105 PMC2587584

[32] Han YH, Moon HJ, You BR, Kim SZ, Kim SH, Park WH. Effects of carbonyl cyanide p-(trifluoromethoxy) phenylhydrazone on the growth inhibition in human pulmonary adenocarcinoma Calu-6 cells. Toxicology. 2009;265:101–7. 19819288 10.1016/j.tox.2009.10.001

[33] Lim MLR, Minamikawa T, Nagley P. The protonophore CCCP induces mitochondrial permeability transition without cytochrome *c* release in human osteosarcoma cells. FEBS Lett. 2001;503:69–74. 11513857 10.1016/s0014-5793(01)02693-x

[34] Satoh K, Zhang L, Zhang Y, Chelluri R, Boufraqech M, Nilubol N, et al. Identification of niclosamide as a novel anticancer agent for adrenocortical carcinoma. Clin Cancer Res. 2016;22:3458–66. 26873959 10.1158/1078-0432.CCR-15-2256 PMC4947455

[35] Schweizer MT, Haugk K, McKiernan JS, Gulati R, Cheng HH, Maes JL, et al. A phase I study of niclosamide in combination with enzalutamide in men with castration-resistant prostate cancer. PLoS One. 2018;13:e0198389. 29856824 10.1371/journal.pone.0198389 PMC5983471

[36] Parikh M, Liu C, Wu C-Y, Evans CP, Dall’Era M, Robles D, et al. Phase Ib trial of reformulated niclosamide with abiraterone/prednisone in men with castration-resistant prostate cancer. Sci Rep. 2021;11:6377. 33737681 10.1038/s41598-021-85969-x PMC7973745

[37] Nascimento-Gonçalves E, Mendes BAL, Silva-Reis R, Faustino-Rocha AI, Gama A, Oliveira PA. Animal models of colorectal cancer: from spontaneous to genetically engineered models and their applications. Vet Sci. 2021;8:59. 33916402 10.3390/vetsci8040059 PMC8067250

[38] Becker C. In vivo imaging of colitis and colon cancer development in mice using high resolution chromoendoscopy. Gut. 2005;54:950–4. 15951540 10.1136/gut.2004.061283 PMC1774595

[39] Stolfi C, De Simone V, Colantoni A, Franzè E, Ribichini E, Fantini MC, et al. A functional role for Smad7 in sustaining colon cancer cell growth and survival. Cell Death Dis. 2014;5:e1073–e1073. 24556688 10.1038/cddis.2014.49 PMC3944263

[40] Sato T, Stange DE, Ferrante M, Vries RGJ, van Es JH, van den Brink S, et al. Long-term expansion of epithelial organoids from human colon, adenoma, adenocarcinoma, and Barrett’s epithelium. Gastroenterology. 2011;141:1762–72. 21889923 10.1053/j.gastro.2011.07.050

