# Supplementary figures and images for: Rafoxanide disrupts mitochondrial homeostasis through VDAC1 modulation in colorectal cancer cells
Source: Cell Death Discov. 2026 Mar 5;12:142. doi: 10.1038/s41420-026-02986-3 (PMC13039284; doi:10.1038/s41420-026-02986-3)

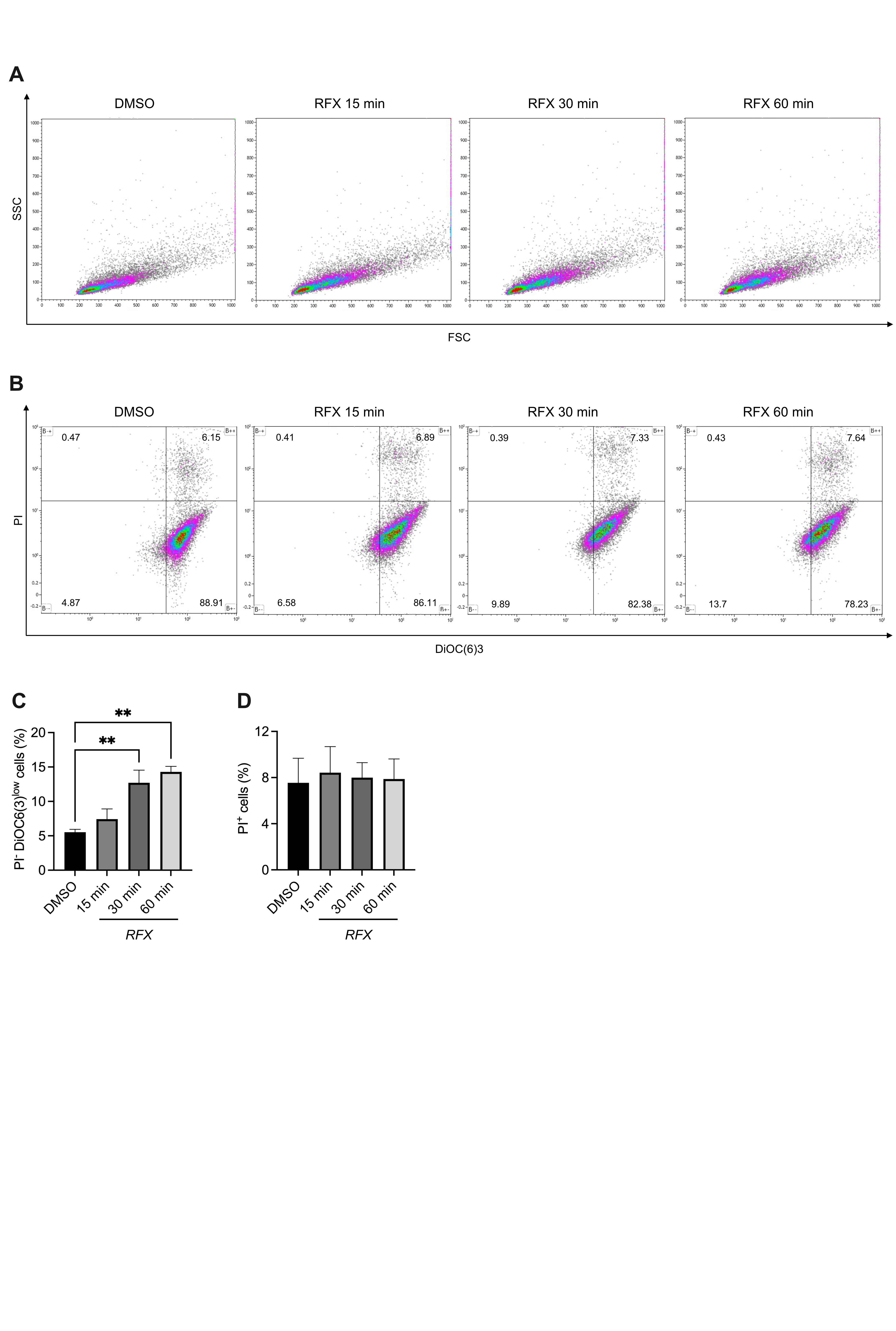

Supplement: Supplementary file 1 — Suppl. Figure 1 [file 41420_2026_2986_MOESM1_ESM.png]

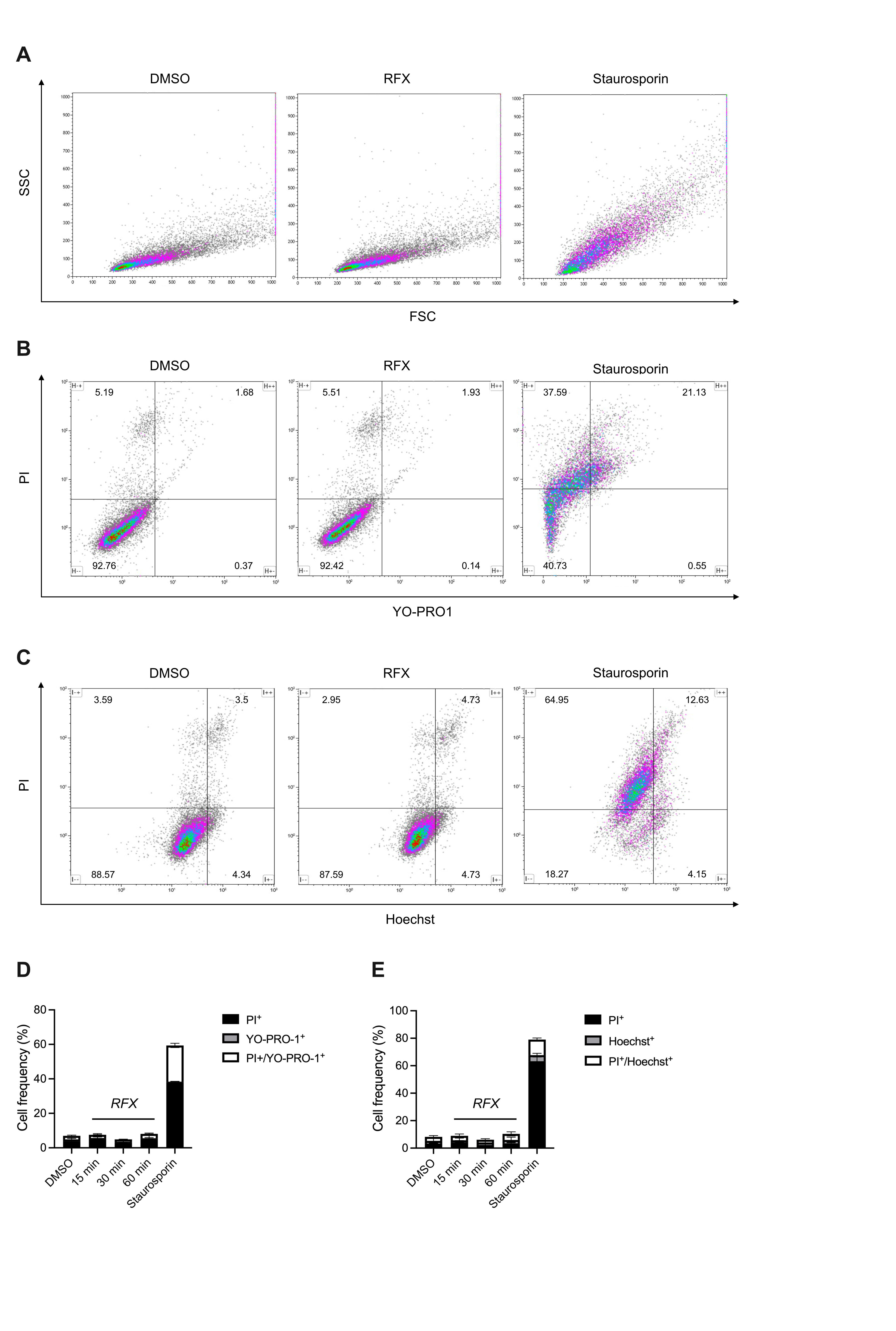

Supplement: Supplementary file 2 — Suppl. Figure 2 [file 41420_2026_2986_MOESM2_ESM.png]

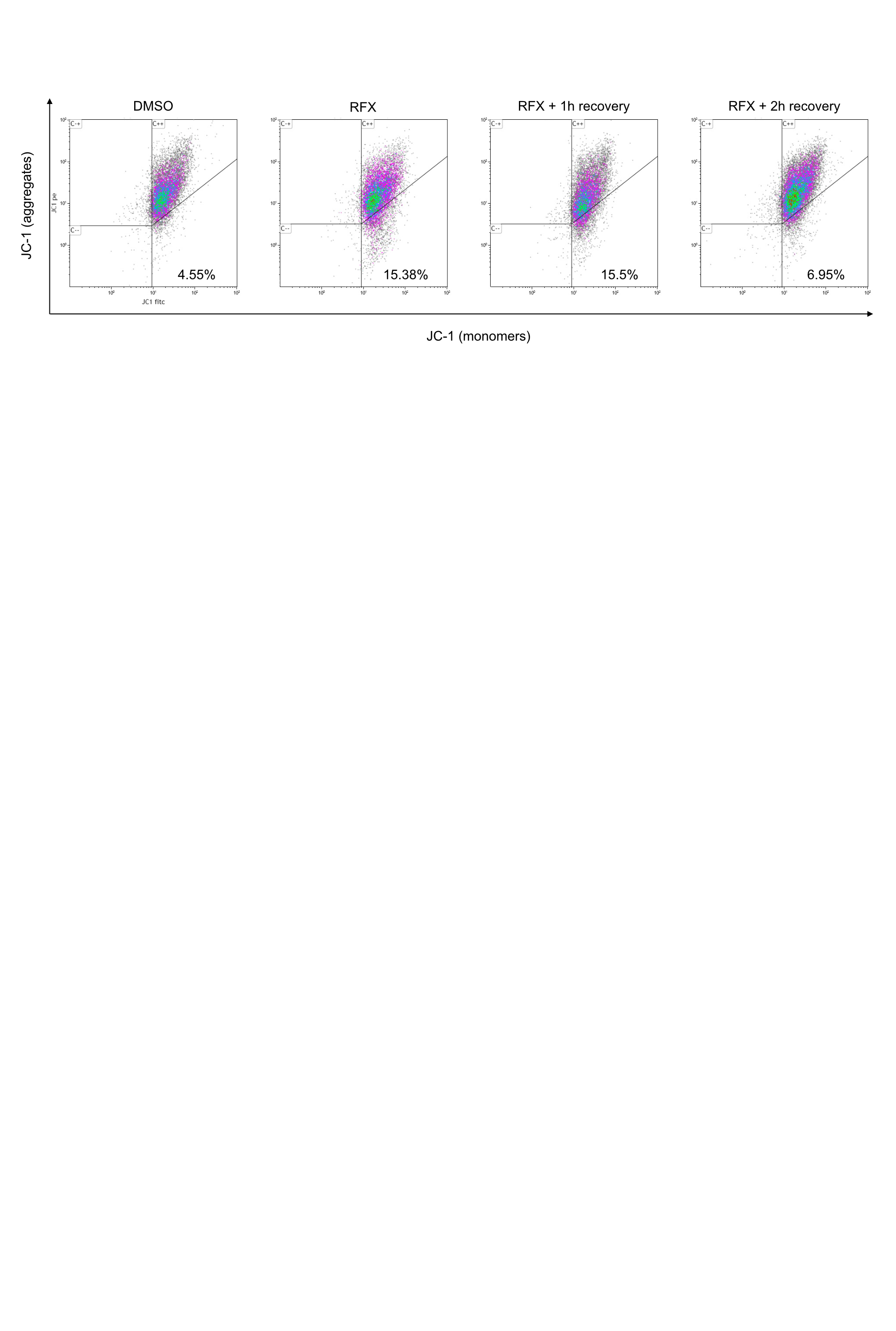

Supplement: Supplementary file 3 — Suppl. Figure 3 [file 41420_2026_2986_MOESM3_ESM.png]

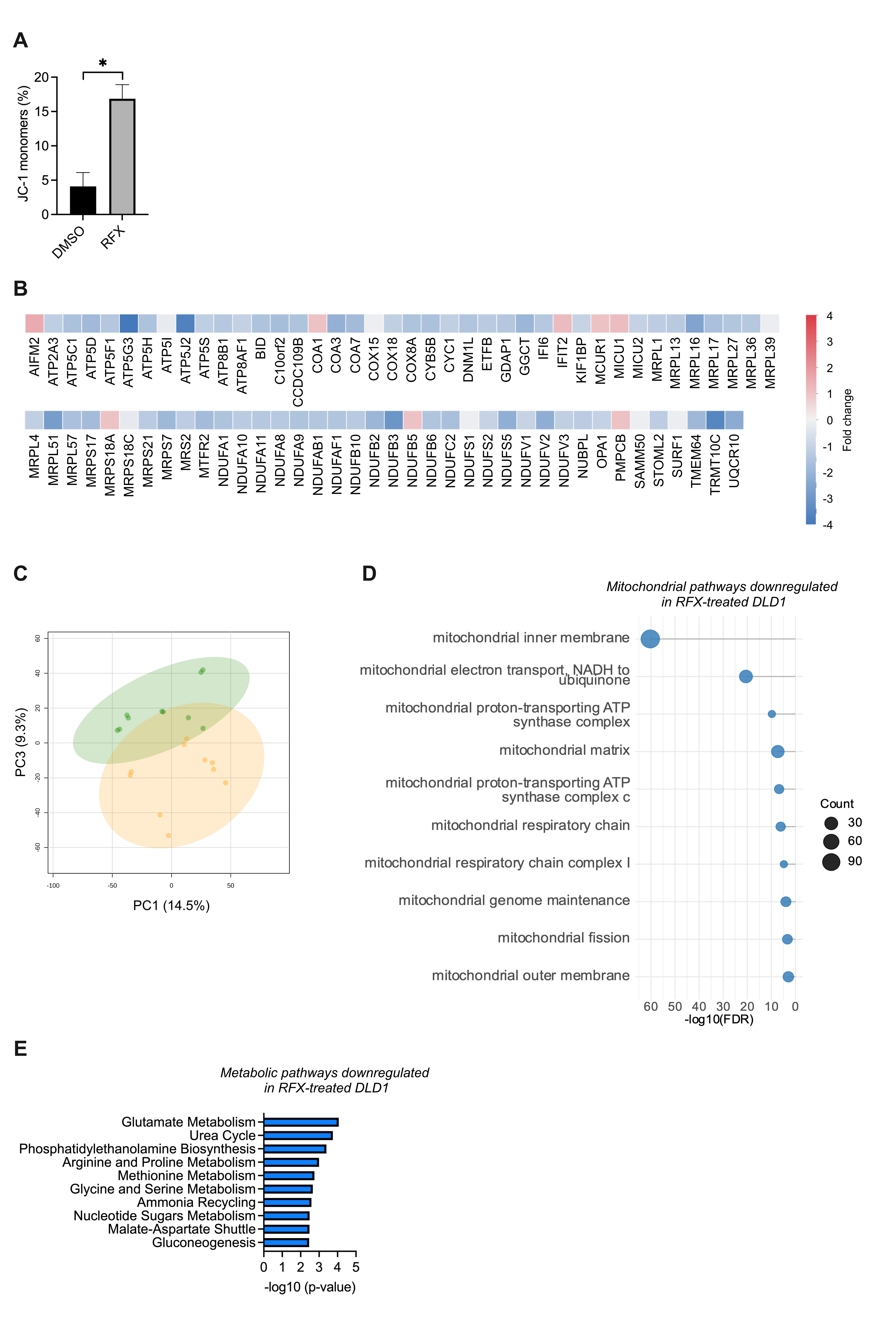

Supplement: Supplementary file 4 — Suppl. Figure 4 [file 41420_2026_2986_MOESM4_ESM.png]

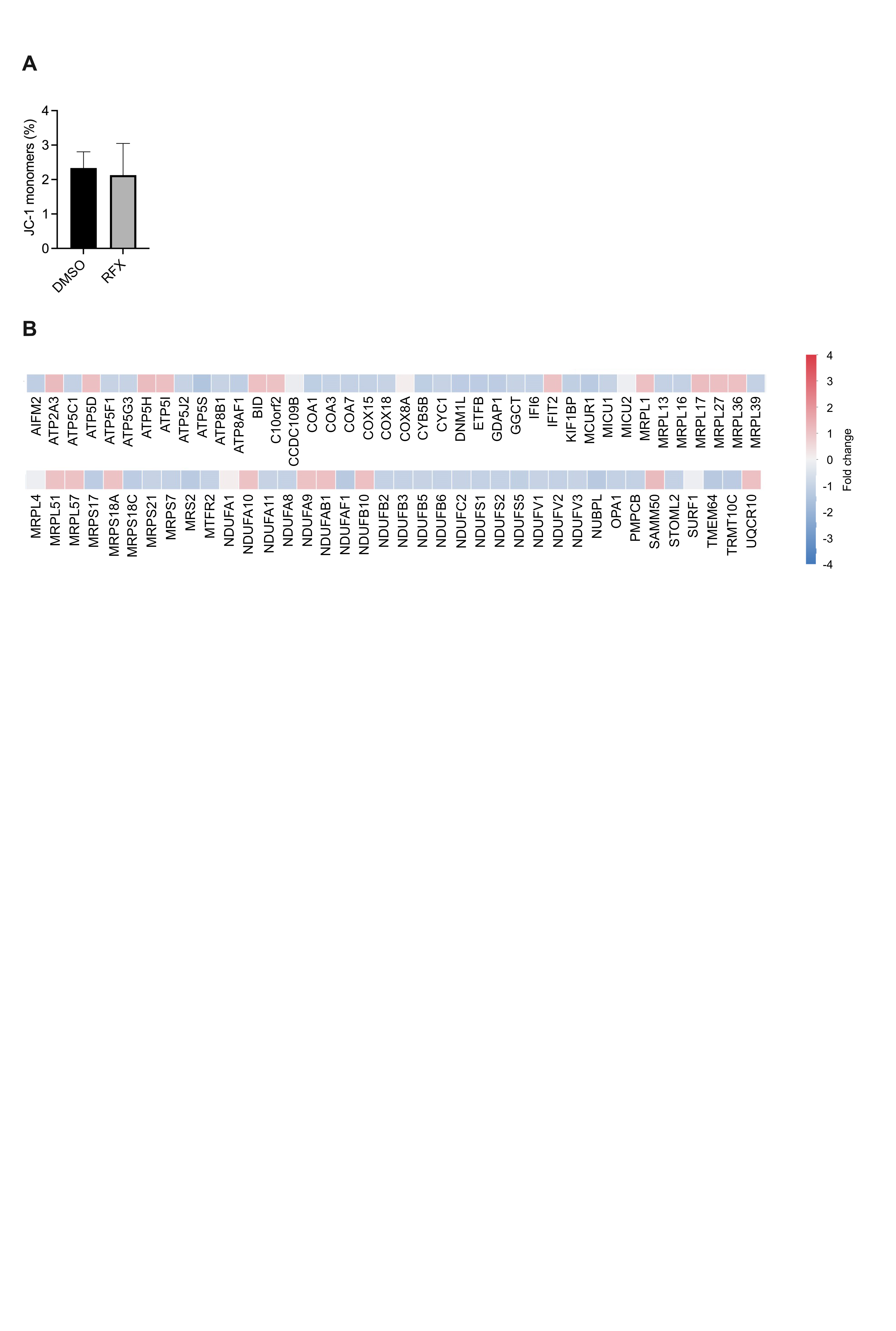

Supplement: Supplementary file 5 — Suppl. Figure 5 [file 41420_2026_2986_MOESM5_ESM.png]

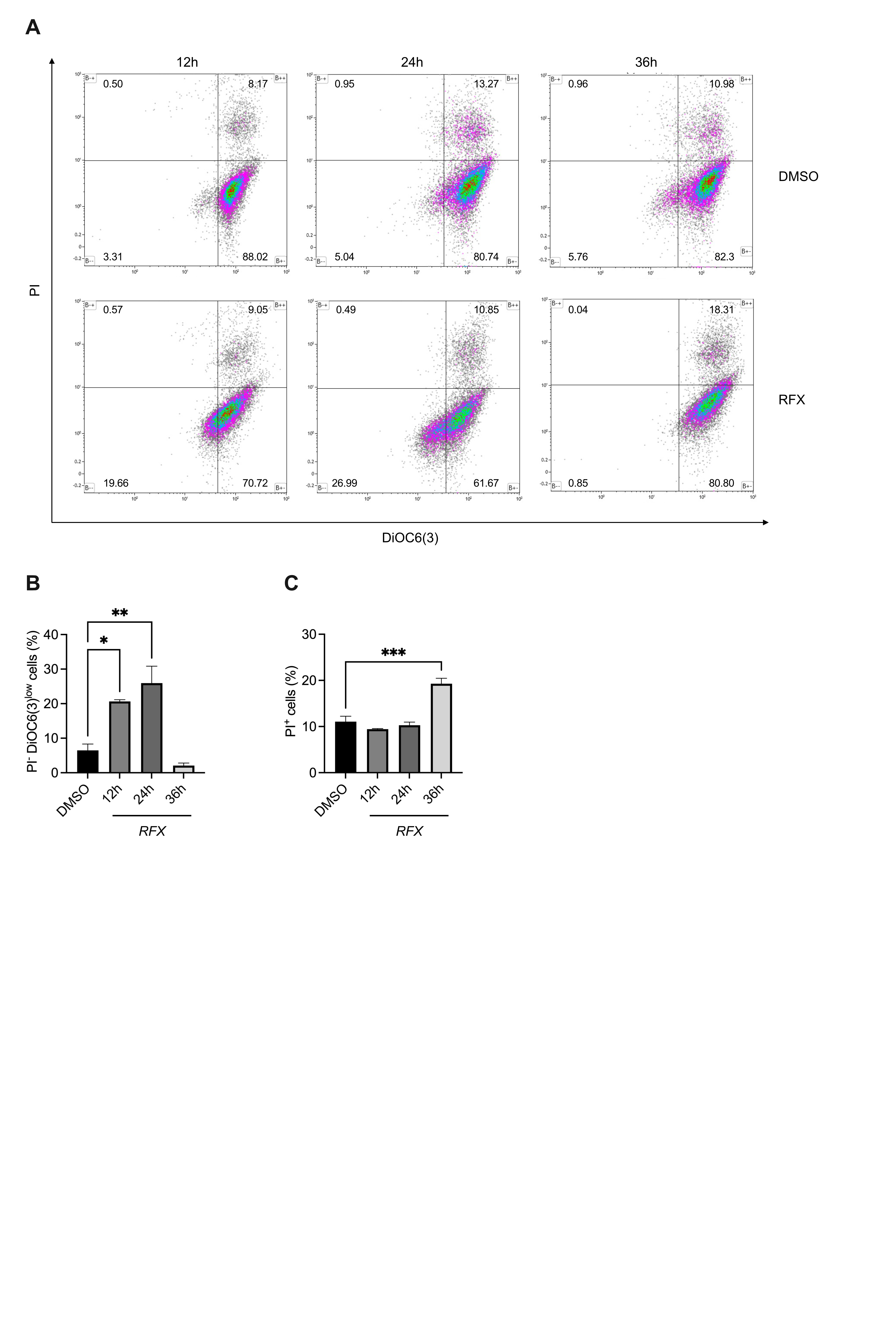

Supplement: Supplementary file 6 — Suppl. Figure 6 [file 41420_2026_2986_MOESM6_ESM.png]

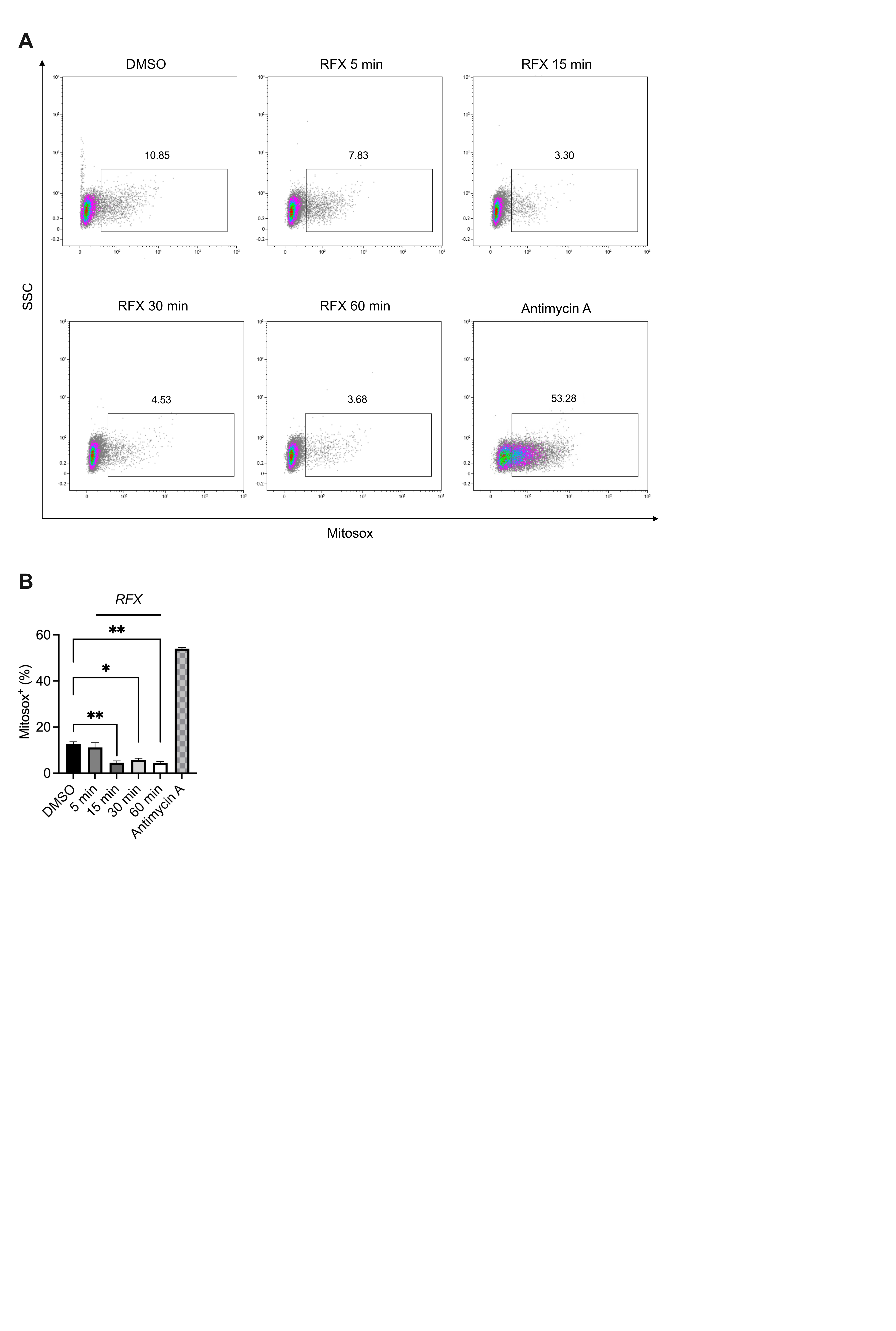

Supplement: Supplementary file 7 — Suppl. Figure 7 [file 41420_2026_2986_MOESM7_ESM.png]

Figure 4

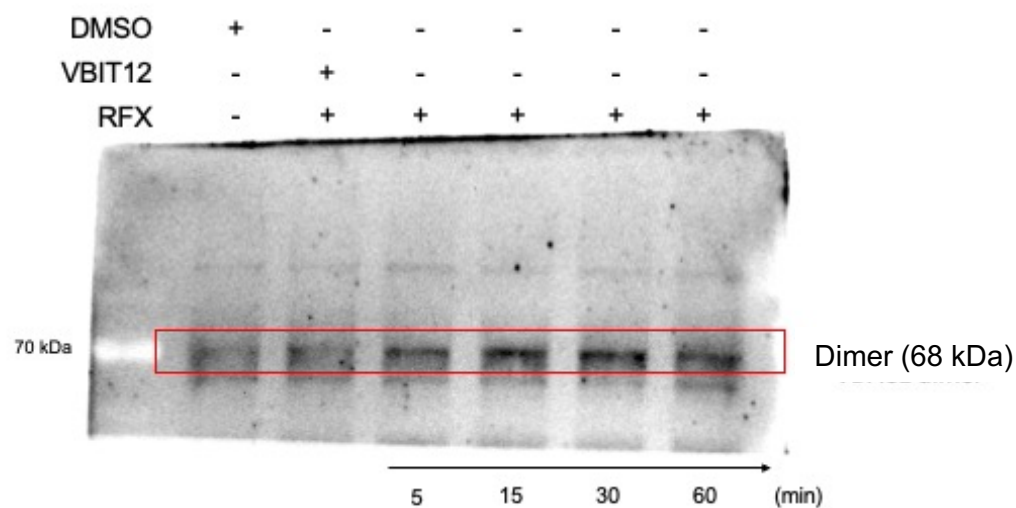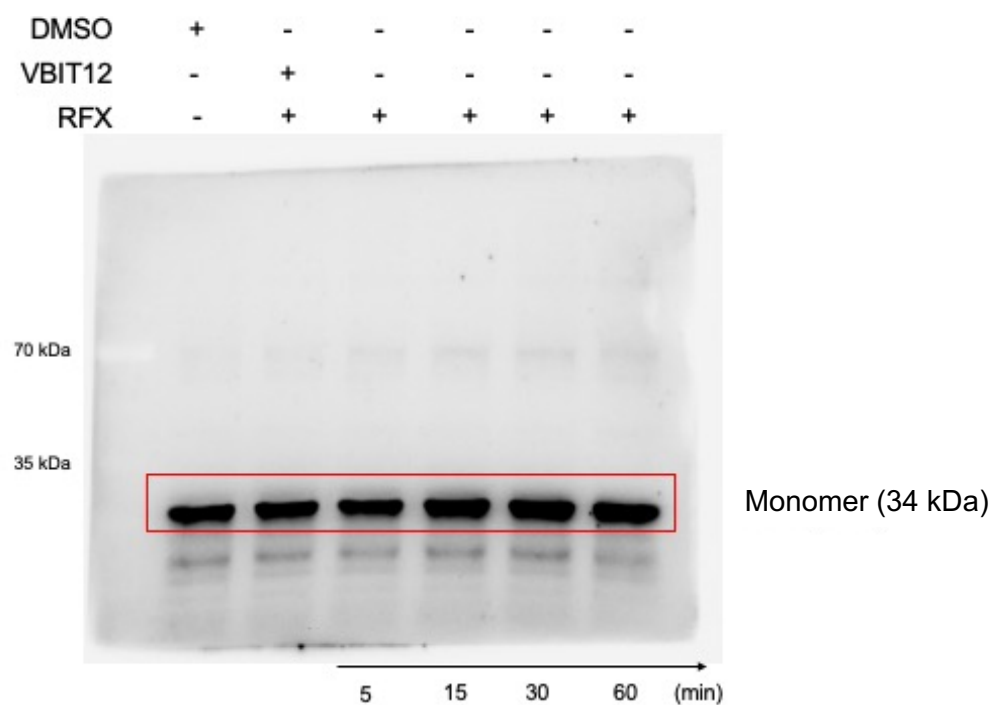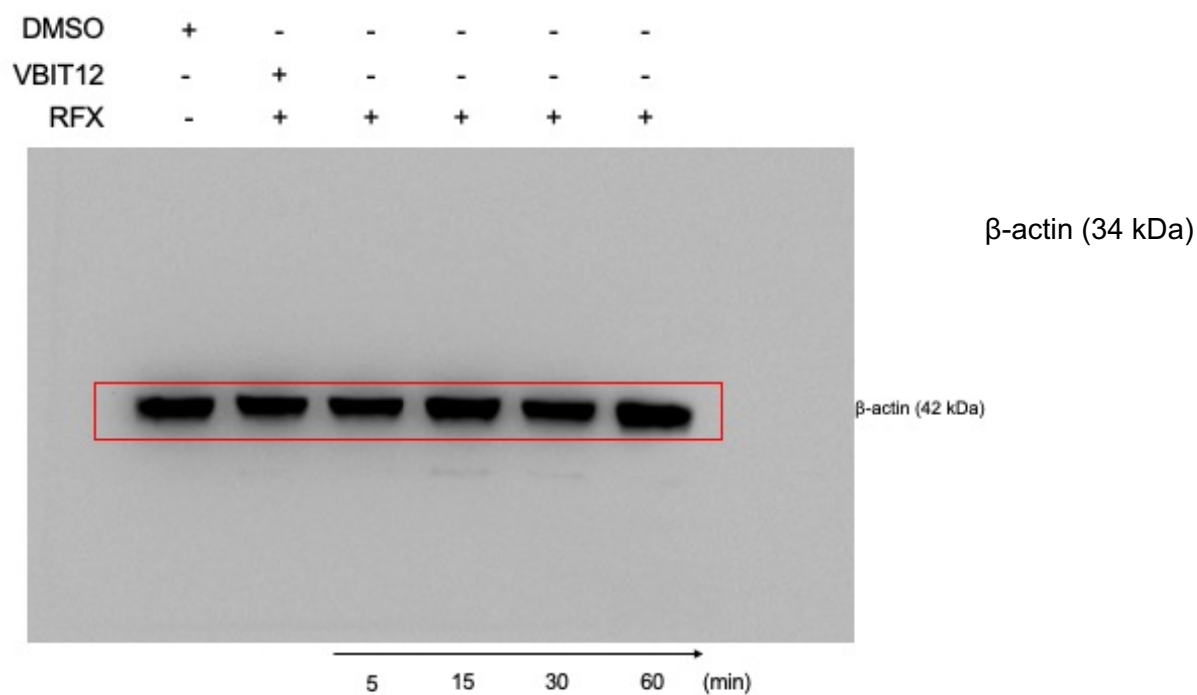

|      |   |   |   |
|------|---|---|---|
| DMSO | + | - | - |
| RFX  | - | + | + |
| NAC  | - | - | + |

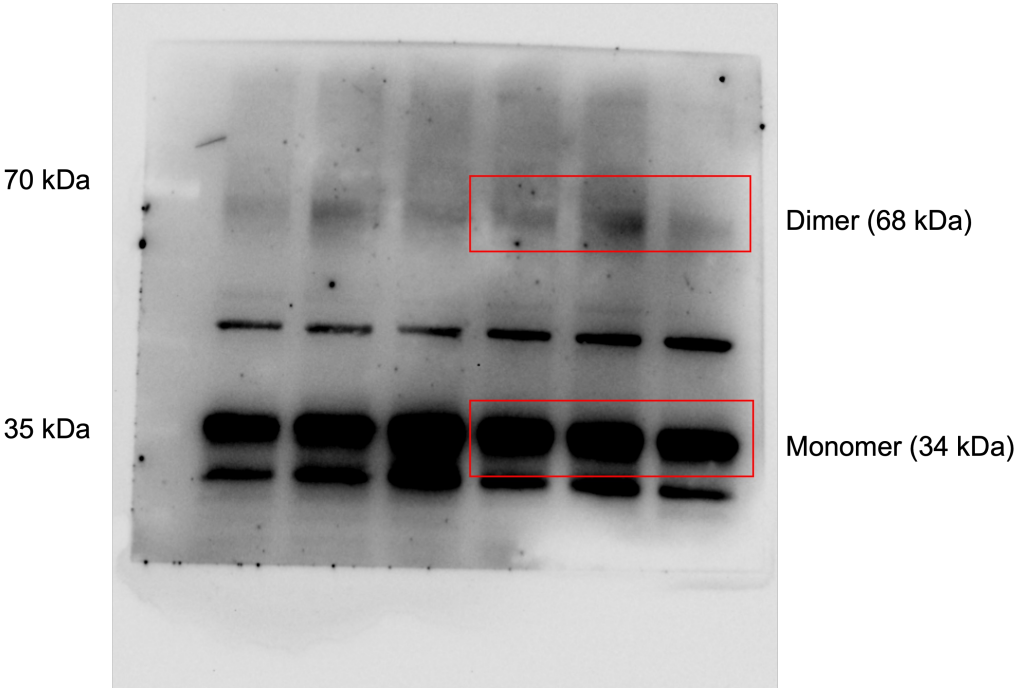

|      |   |   |   |
|------|---|---|---|
| DMSO | + | - | - |
| RFX  | - | + | + |
| NAC  | - | - | + |

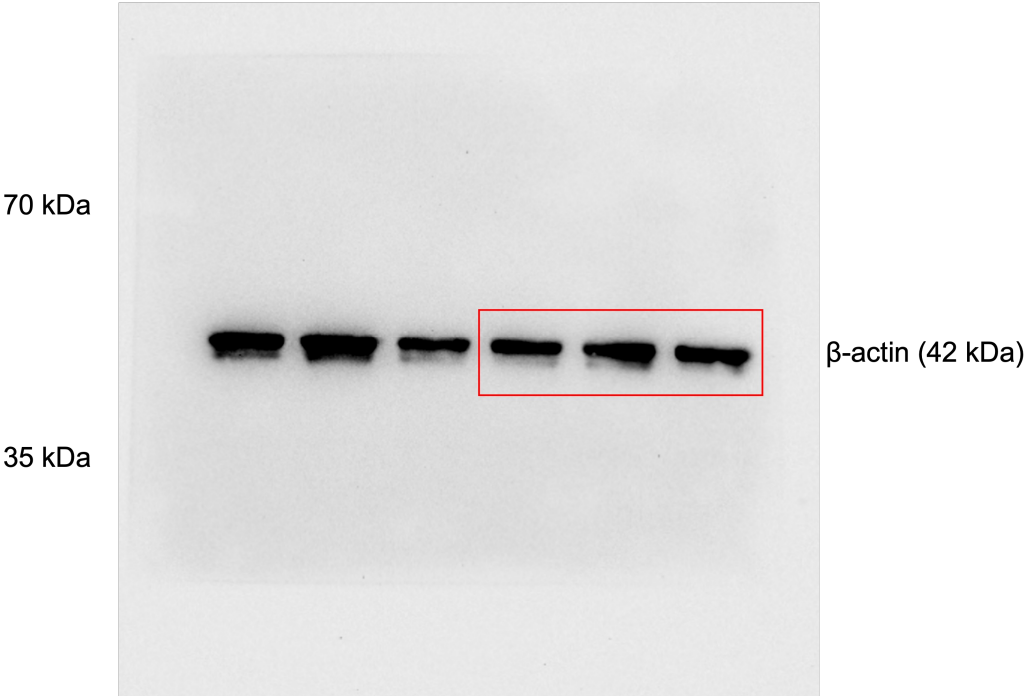

Figure 3A

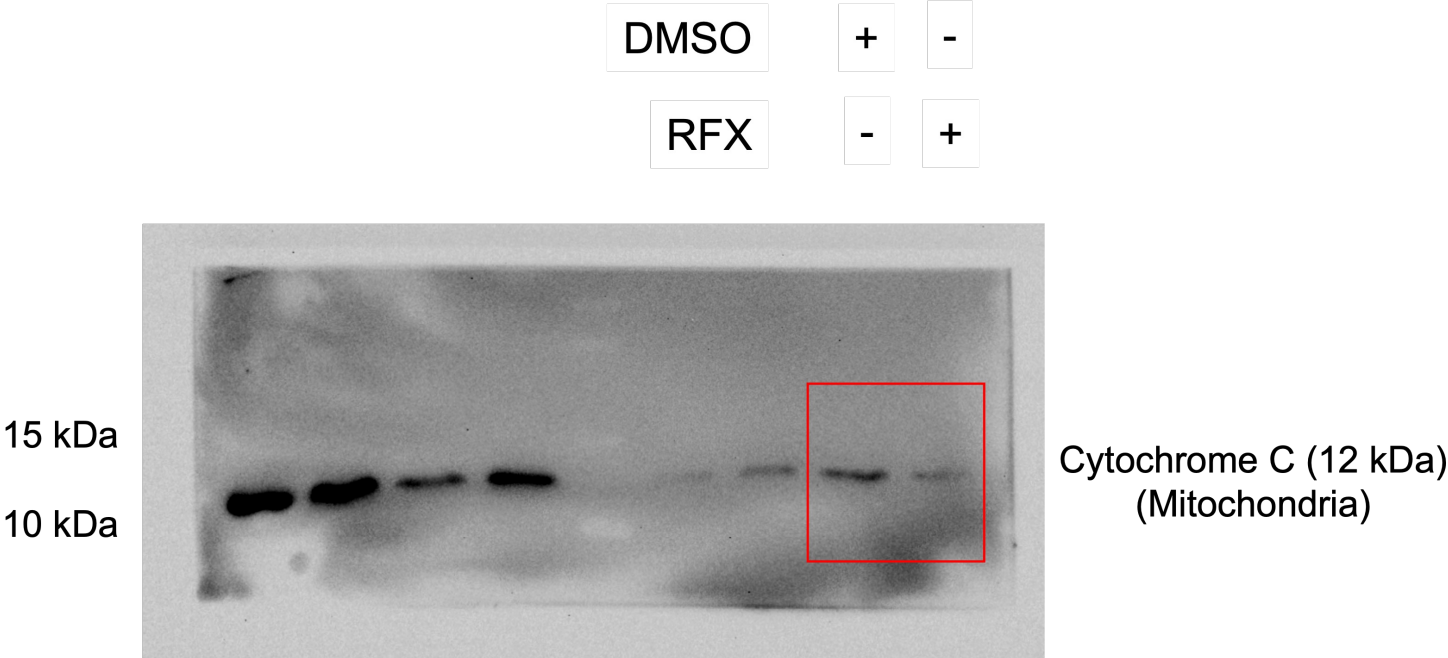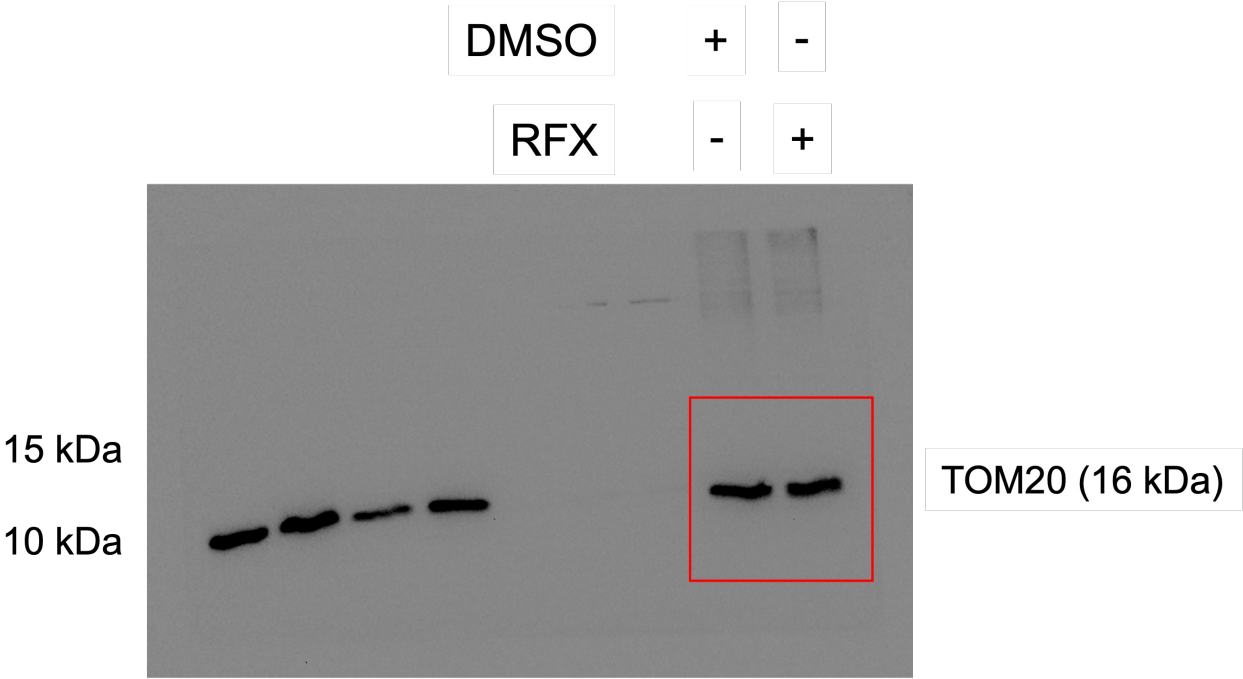

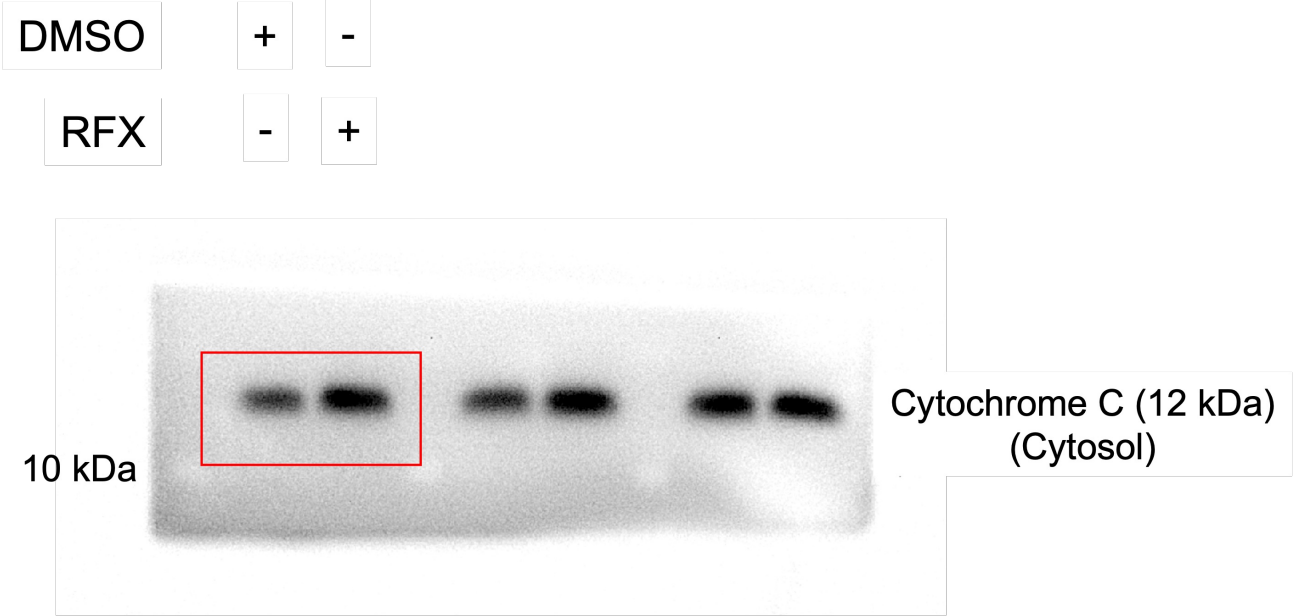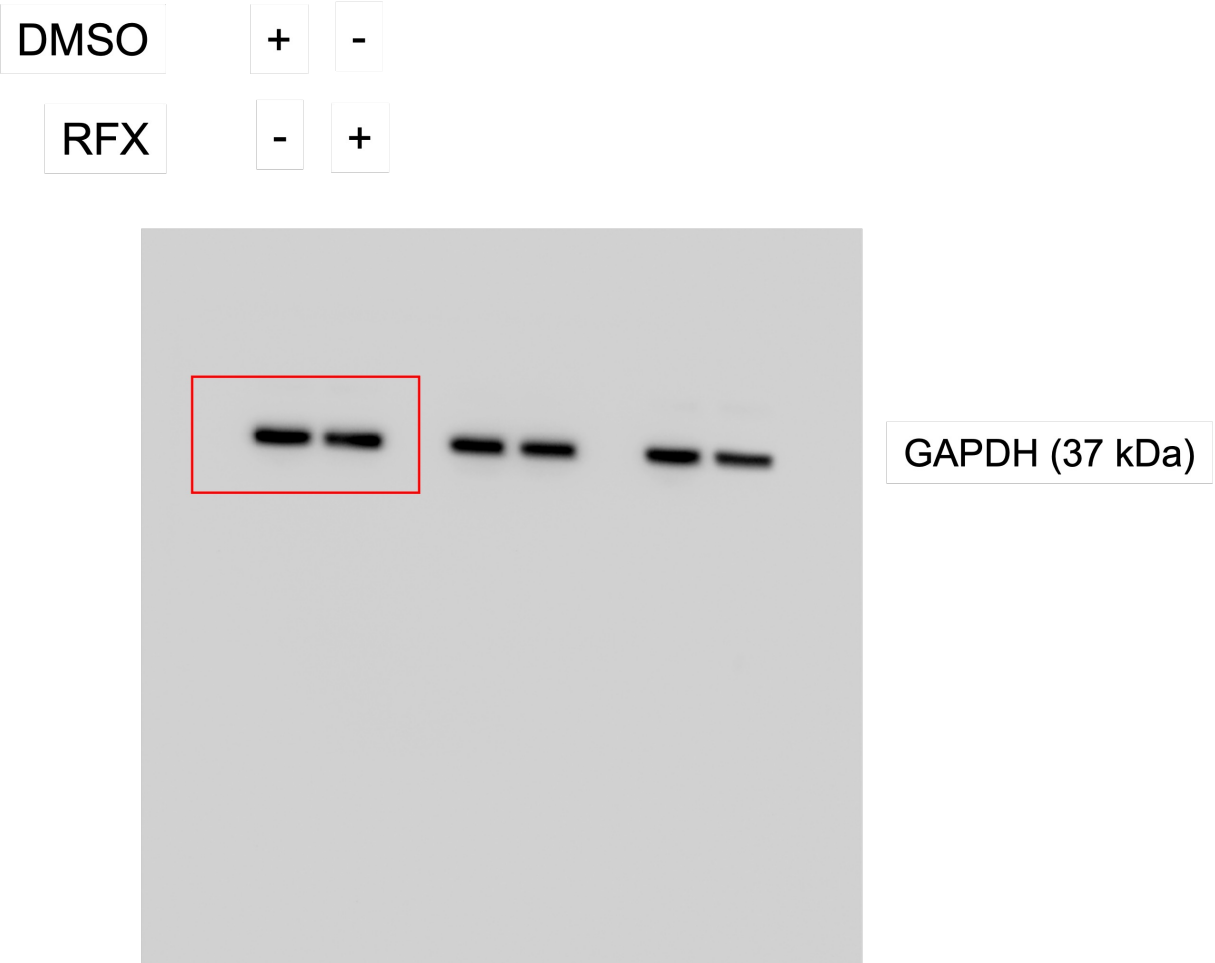

Supplement: Supplementary file 10 — uncropped wb [file 41420_2026_2986_MOESM10_ESM.pdf]
