# Supplementary material for: Rafoxanide disrupts mitochondrial homeostasis through VDAC1 modulation in colorectal cancer cells
Source: Cell Death Discov. 2026 Mar 5;12:142. doi: 10.1038/s41420-026-02986-3 (PMC13039284; doi:10.1038/s41420-026-02986-3)
Supplement: Supplementary file 8 — Suppl. figure legend [file 41420_2026_2986_MOESM8_ESM.docx]

**Supplementary figure legends**

**Rafoxanide disrupts mitochondrial homeostasis through VDAC1 modulation in colorectal cancer cells.**

Lorenzo Tomassini^1^, Teresa Pacifico^1^, Mattia Alberto Serra^1^, Eduardo Maria Sommella^2^, Manolo Sambucci^3^, Giuseppe S. Sica^4^, Luca Savino^5^, Sara Vitale^6^, Angela Ortenzi^1^, Livia Biancone^1,7^, Luca Battistini^3^, Giovanna Borsellino^3^, Ivan Monteleone^8^, Vincenzo Barnaba^9^, Micol Eleonora Fiori^6^, Giovanni Monteleone^1,7^, Carmine Stolfi^1^, Federica Laudisi^1*^.

^1^ Department of Systems Medicine, University of Rome Tor Vergata, Rome, Italy.

^2^ Department of Pharmacy, University of Salerno, Fisciano, 84084, Salerno, Italy.

^3^ Neuroimmunology Unit, Santa Lucia Foundation IRCCS, Rome, Italy.

^4^ Department of Surgery, University of Rome Tor Vergata, Rome, Italy.

^5^ Department of Integrated Care Processes, University of Rome Tor Vergata, Rome, Italy.

^6^ Department of Oncology and Molecular Medicine, Istituto Superiore di Sanità, Rome, Italy.

^7^ Gastroenterology Unit, Policlinico Universitario Tor Vergata, 00133 Rome, Italy

^8^ Department of Biomedicine and Prevention, University of Rome Tor Vergata, Rome, Italy.

^9^ Istituto Pasteur Italia and Sapienza Università di Roma, Rome, Italy.

**Figure Legends**

**Suppl. Figure 1. Rafoxanide impairs mitochondrial membrane potential in HCT116 cells without affecting cell viability. (A-B)** Representative dot plots from flow cytometry analysis showing forward (FSC) and side scatter (SSC) parameters (**A**) and the frequencies of HCT116 cells expressing high levels (polarized mitochondrial membrane) and low levels (depolarized mitochondrial membrane) of DiOC6(3) dye among propidium iodide (PI)-negative (live) and PI-positive (dead) cells (**B**) treated with rafoxanide (RFX, 2.5 μM) or DMSO (vehicle) for 15, 30, and 60 minutes. Numbers indicate the percentage of cells in the designated quadrants. (**C-D**) Bar plots showing the frequencies of PI-negative and DiOC6(3) low-expressing HCT116 cells (**C**) and PI-positive cells (**D**) treated as indicated in panel **A**. Values are mean ± SEM of 4 independent experiments. Differences among groups were compared using one-way analysis of variance (ANOVA) followed by the Tukey’s post hoc test (* P≤.05).

**Suppl. Figure 2. Short-term rafoxanide treatment does not induce chromatin condensation, plasma membrane permeabilization, or cell death in HCT116 cells. (A-B)** Representative dot plots from flow cytometry analysis showing forward (FSC) and side scatter (SSC) parameters (**A**) and staining with propidium iodide (PI), YO-PRO-1, and Hoechst dyes (**B-C**) in HCT116 cells treated with rafoxanide (RFX, 2.5 μM) or DMSO (vehicle) for 60 minutes. PI-positive cells were considered necrotic, YO-PRO-1–positive cells apoptotic, and Hoechst-positive cells indicative of chromatin condensation. Staurosporin treatment was used as a positive control for cell death. Numbers indicate the percentage of cells in the designated quadrants. (**D-E**) Quantification of the percentage of HCT116 cells positive for PI, YO-PRO-1, or Hoechst staining, as well as dye-negative (viable) cells treated as indicated in panel **A**. Values are mean ± SEM of 4 independent experiments. Differences among groups were compared using one-way analysis of variance (ANOVA) followed by the Tukey’s post hoc test (* P≤.05).

**Suppl. Figure 3.** **Rafoxanide-induced mitochondrial membrane depolarization is reversible upon drug removal.** Representative flow cytometry dot plots showing JC-1 fluorescence in HCT116 cells treated with rafoxanide (RFX, 2.5 μM) or vehicle (DMSO) for 1 hour and subsequently transferred to fresh medium for an additional 1 or 2 hours. JC-1 aggregates indicate polarized mitochondria, whereas JC-1 monomers indicate mitochondrial membrane depolarization.

**Suppl. Figure 4. Prolonged rafoxanide treatment induces mitochondrial dysfunction in DLD1 cells.** (**A**) Quantification of mitochondrial membrane depolarization assessed by JC-1 staining in DLD1 cells treated with rafoxanide (RFX, 2.5 μM) or DMSO (vehicle) for 1 hour. Values are mean ± SEM of 3 independent experiments. Differences were compared using a two-tailed Student’s t-test (* P≤.05). (**B**) Heatmap showing differentially expressed mitochondria-associated genes ranked by log2 fold change (log2FC). Results were obtained from the comparison between DLD1 cells treated with rafoxanide (RFX, 2.5 μM) or DMSO (vehicle) for 24 hours. (**C**) Principal Component Analysis (PCA) representation of the proteomic profile of DLD1 cells treated with rafoxanide (orange) or DMSO (green) for 24 hours. (**D**) Lollipop plot showing the top 10 downregulated proteomic pathways ranked by -log10 false discovery rate (-log10FDR), calculated on the set of significantly downregulated mitochondrial proteins in DLD1 cells treated as indicated in panel **C**. (**E**) Metabolic pathway enrichment analysis performed using SMPDB (The Small Molecule Pathway Database) on the set of significantly downregulated metabolites identified in DLD1 cells treated as indicated in panel **C**.

**Suppl. Figure 5. Rafoxanide does not impair mitochondrial function in non-tumor colonic epithelial cells.**

Quantification of mitochondrial membrane depolarization assessed by JC-1 staining in HCEC-1CT cells treated with rafoxanide (RFX, 2.5 μM) or DMSO (vehicle) for 1 hour. Values are mean ± SEM of 3 independent experiments. (**B**) Heatmap showing differentially expressed mitochondria-associated genes ranked by log2 fold change (log2FC). Results were obtained from the comparison between HCEC-1CT cells treated with rafoxanide (RFX, 2.5 μM) or DMSO (vehicle) for 24 hours.

**Suppl. Figure 6. Prolonged rafoxanide treatment induces mitochondrial depolarization and cell death in HCT116 cells.** Representative flow cytometry dot plots showing the frequencies of HCT116 cells expressing high levels (polarized mitochondrial membrane) and low levels (depolarized mitochondrial membrane) of DiOC6(3) dye among propidium iodide (PI)-negative (live) and PI-positive (dead) cells treated with rafoxanide (RFX, 2.5 μM) or DMSO (vehicle) for 12, 24, and 36 hours. Numbers indicate the percentage of cells in the designated quadrants. (**B-C**) Bar plots showing the frequencies of PI-negative and DiOC6(3) low-expressing HCT116 cells (**B**) and PI-positive cells (**C**) treated as indicated in panel **A**. Values are mean ± SEM of 4 independent experiments. Differences among groups were compared using one-way analysis of variance (ANOVA) followed by the Tukey’s post hoc test (* P≤.05).

**Suppl. Figure 7. Rafoxanide reduces mitochondrial superoxide production in HCT116 cells.** (**A-B**) Representative flow cytometry dot plots (**A**) and quantitative bar graphs (**B**) showing the percentage of MitoSOX positive cells treated with rafoxanide (RFX, 2.5 μM) or DMSO (vehicle) for 15, 30, and 60 minutes. MitoSOX fluorescence was used as a readout of mitochondrial superoxide production. Treatment with antimycin A was used as a positive control for mitochondrial ROS induction. Numbers indicate the percentage of cells in the designated quadrants. Values are mean ± SEM of 3 independent experiments. Differences among groups were compared using one-way analysis of variance (ANOVA) followed by the Tukey’s post hoc test (* P≤.05).
