# Supplementary material for: Rafoxanide disrupts mitochondrial homeostasis through VDAC1 modulation in colorectal cancer cells
Source: Cell Death Discov. 2026 Mar 5;12:142. doi: 10.1038/s41420-026-02986-3 (PMC13039284; doi:10.1038/s41420-026-02986-3)
Supplement: Supplementary file 9 — Suppl. materials&methods [file 41420_2026_2986_MOESM9_ESM.docx]

**Supplementary materials**

**Rafoxanide disrupts mitochondrial homeostasis through VDAC1 modulation in colorectal cancer cells**

Lorenzo Tomassini^1^, Teresa Pacifico^1^, Mattia Alberto Serra^1^, Eduardo Maria Sommella^2^, Manolo Sambucci^3^, Giuseppe S. Sica^4^, Luca Savino^5^, Sara Vitale^6^, Angela Ortenzi^1^, Livia Biancone^1,7^, Luca Battistini^3^, Giovanna Borsellino^3^, Ivan Monteleone^8^, Vincenzo Barnaba^9^, Micol Eleonora Fiori^6^, Giovanni Monteleone^1,7^, Carmine Stolfi^1^, Federica Laudisi^1*^.

^1^ Department of Systems Medicine, University of Rome Tor Vergata, Rome, Italy.

^2^ Department of Pharmacy, University of Salerno, Fisciano, 84084, Salerno, Italy.

^3^ Neuroimmunology Unit, Santa Lucia Foundation IRCCS, Rome, Italy.

^4^ Department of Surgery, University of Rome Tor Vergata, Rome, Italy.

^5^ Department of Integrated Care Processes, University of Rome Tor Vergata, Rome, Italy.

^6^ Department of Oncology and Molecular Medicine, Istituto Superiore di Sanità, Rome, Italy.

^7^ Gastroenterology Unit, Policlinico Universitario Tor Vergata, 00133 Rome, Italy

^8^ Department of Biomedicine and Prevention, University of Rome Tor Vergata, Rome, Italy.

^9^ Istituto Pasteur Italia and Sapienza Università di Roma, Rome, Italy.

**Materials & Methods**

**1. Immunohistochemistry**

Cryosections of colonic tumor samples isolated from Balb/c mice were stained with a primary antibody directed against Ki‐67 (1:100, #M7249, Dako, Glostrup, Denmark). Positive cells were visualized using the MACH4 Universal HRP Polymer kit with DAB (Biocare Medical #M4BD534G, Pacheco, California, USA) and analyzed with the Leica DMI4000 B microscope using the Leica Application Suite Software (version 4.6.2).

**2. Immunofluorescence**

HCT-116 cells were seeded at approximately 1.5 x 10^5 in a 6-well plate on a microscope slide and stimulated with rafoxanide (2.5 μM) or vehicle (DMSO) overnight. Cells were then fixed with 4% PFA for 10 minutes, washed three times with PBS, and permeabilized with Tryton-X 0.1% for 20 minutes. A blocking procedure (bovine serum albumin 1%, goat serum 10%) was performed for 1 hour at room temperature, and rabbit primary antibody against cytochrome C (1:300, #4280, Cell Signaling Technology, Danvers, Massachusetts, USA) was incubated overnight at 4°C. After washing with PBS, the secondary antibody (1:2000, #A11008; Invitrogen, Waltham, Massachusetts, USA) was applied for 2 hours at room temperature. Slides were mounted using Prolong gold antifade reagent with 4′,6-diamidino-2-phenylindole (#P36931; Invitrogen) and analyzed by a Leica DMI4000 B microscope with Leica (Wetzlar, Germany) application suite software (V4.6.2).

**3. Western blot analysis**

HCT116 colorectal cancer cells were seeded in 100-mm culture dishes at a density of 4 × 10^6 cells and treated with rafoxanide (2.5 μM) or vehicle (DMSO). Mitochondria were then isolated using the Mitochondria Isolation Kit for Cultured Cells (#89874, Thermo Fisher Scientific, Waltham, Massachusetts, USA), according to the manufacturer’s instructions. Both mitochondrial and cytosolic fractions were collected and subjected to protein extraction using a lysis buffer containing 10 mM HEPES (pH 7.9), 10 mM potassium chloride (KCl), 0.1 mM ethylenediaminetetraacetic acid (EDTA), 0.2 mM ethylene glycol-bis (β-aminoethyl ether)-N,N,N’, N’-tetraacetic acid (EGTA), and 0.5% Nonidet P40 supplemented with 1 mM dithiothreitol [DTT], 10 mg/ml aprotinin, 10 mg/ml leupeptin, 1 mM phenylmethylsulphonyl fluoride (PMSF), 1 mM Na3VO4, and 1 mM sodium fluoride (NaF). Lysates were clarified by centrifugation and separated on sodium dodecyl sulphate (SDS) polyacrylamide gel electrophoresis. Blots were incubated with antibodies against cytochrome c (1:500, #sc-13156, Santa Cruz Biotechnologies), TOM20 (1:1000, #42406, Cell Signaling Technology), and GAPDH (1:1000, #5174, Cell Signaling Technology), followed by a secondary antibody conjugated to horseradish peroxidase (Dako).

**4. Seahorse Assay**

Real-time measurement of Oxygen Consumption Rate (OCR) was made using an XFe-96 Extracellular Flux Analyzer (Seahorse Bioscience-Agilent, Santa Clara, CA, USA). HCT116 and DLD1 cells were plated in XFe-96 plates (Agilent Technologies) at a seeding density of 1.5 x 10^5/ml and treated with rafoxanide (2.5 μM) or vehicle (DMSO) for 15, 30, 60 minutes. OCR was measured in a medium (XF RPMI pH 7.4, Agilent Technologies) supplemented with 10 mM glucose, 2 mM L-glutamine and 1 mM pyruvate, under basal conditions and in response to 2.5 mM oligomycin, 2.0 mM of carbonyl cyanide-4-(trifluoromethoxy) phenylhydrazone (FCCP), and 0.5 mM of Antimycin-A and Rotenone (all reagents contained in the XF Cell Mito Stress Test kit, Agilent Technologies #103015-100) according to the manufacturer’s instructions. Basal OCR was determined before injections of oligomycin, maximal respiratory capacity was calculated as the difference between the highest rate of oxygen consumption measured after adding FCCP and subtracting the non-mitochondrial oxygen consumption. The non-mitochondrial OCR is the rate that remains after the complete shutdown of the mitochondrial electron transport chain by the combined action of rotenone/antimycin-A. Each sample was plated in five replicates.

**5. Complex I and complex III activity assay**

The activity of mitochondrial Complex I was measured in HCT116 cells treated with rafoxanide (2.5 μM) or vehicle (DMSO) using the Complex I Enzyme Activity Microplate Assay Kit (Abcam, ab109721). Briefly, cells were lysed and incubated on ice for 30 minutes. The lysates were centrifuged at 16,000 × g for 20 minutes at 4°C, and the supernatant was collected. Total protein concentration was determined using the Bradford assay, and samples were diluted to a final concentration of 0.5 μg/μL. Subsequently, samples were plated into a 96-well microplate pre-coated with anti-Complex I antibody and incubated at room temperature for 3 hours. Wells were then washed twice, and Assay Solution containing NADH and the chromogenic dye was added. Complex I activity was determined by monitoring the increase in absorbance at 450 nm over time using the DXT880 Multimode reader (Beckman Coulter, Brea, California, USA). The rate of increase in absorbance was normalized to the amount of protein loaded per well.

The activity of mitochondrial Complex III was measured using the Mitochondrial Complex III Activity Assay Kit (Abcam, ab287844). Isolated mitochondria were obtained using a commercial mitochondria isolation kit (#89874, Thermo Fisher Scientific). Protein concentration was determined using the Bradford assay, and samples were normalized accordingly. Subsequently, samples were plated into a 96-well microplate and assay solution was added. Samples were incubated at room temperature, and absorbance was recorded at 550 nm every minute for 10 minutes using a multimode reader Tecan Spark 10M (Tecan Austria GmbH, Grödig, Austria). The Complex III specific activity and Net Complex III activity were calculated and normalized to the total protein content per sample.

**6. Transcriptomic analysis**

HCT116, DLD1 and HCEC-1CT cells were treated or not with rafoxanide for 24 hours, and cell pellets were collected. Total RNA was extracted using the RNeasy Mini Kit (#74104, Qiagen, Hilden, Germany) and digested with DNase (#EN0521, Thermo Fisher Scientific). Samples with quantified complementary DNA were sequenced in the Microarray Unit of the Consortium for Genomic Technologies (Milan, Italy) by hybridization to Affymetrix Human GeneChip Clariom S microarrays. Transcripts were ranked by log2 fold change (log2FC) generated from the comparison between HCT116 treated with rafoxanide (RFX) vs control (DMSO), DLD1 treated with rafoxanide (RFX) vs control (DMSO), and HCEC-1CT treated with rafoxanide (RFX) vs control (DMSO),

**7. Proteomic analysis**

HCT116 and DLD1 cells were treated with rafoxanide (2.5 μM) or vehicle (DMSO) for 24 hours, while human CRC explants and patient-derived organoids were stimulated with rafoxanide (10 μM) or vehicle (DMSO) overnight and for 24 hours, respectively. Samples were then collected, lysed and digested by SONDA S.r.l. (Salerno, Italy). The resulting peptides were subjected to clean-up and purification with in-stage tips using iST kits from PREOMICS (Preomics, Munich, Germany), then analyzed by label-free quantification proteomics. Analyses were performed by nLC-HRMS using an Ultimate 3000 nanoLC (Thermo Fisher Scientific) coupled to an Orbitrap Lumos tribrid mass spectrometer with an Easy nano electrospray ion source (all from Thermo Fisher Scientific). Peptides were trapped for 1 minute in a PepMap trap-Cartridge, 100 Å, 5 µm, 0.3 x 5 mm (Thermo Fisher Scientific), and separated onto a C18-reversed phase column (250 mm × 75 μm I.D, 2.0 µm, 100Å, Thermo Fisher Scientific) with a linear gradient over 60 min. Mobile phases were A): 0,1% HCOOH in water v/v; B): 0,1% HCOOH in ACN/Water v/v 80/20. HRMS analysis was performed in data-dependent acquisition (DDA).

**8. Metabolomic analysis**

HCT116 and DLD1 cells were treated with rafoxanide (2.5 μM) or vehicle (DMSO) for 24 hours or not, and cell pellets were collected and sent to SONDA S.r.l. (Salerno, Italy). Samples were dried with a SpeedVac (Savant, Thermo Fisher Scientific) and resuspended for LC-MS analysis in 50 microliters of 70/30 ACN/H2O. Metabolome analyses were performed on a Vanquish Flex UHPLC coupled online to an Exploris 120 hybrid quadrupole Orbitrap mass spectrometer (Thermo Fisher Scientific) equipped with a heated electrospray ionization probe (HESIII). Metabolome separation was carried out with a BEHA mide column (100×2.1mm; 1.7μm) protected with a Vanguard precolumn (5×2.1mm;1.7μm) (Waters, Milan, Italy). MS1 acquisition was performed at 60.000 resolution, while MS/MS at 15.000 resolution in DDA mode. For the assessment of repeatability and instrument stability overtime, a QC strategy was applied. A quality control (QC) sample was prepared by pooling the same aliquot (10 μl) from each sample. Samples were injected in randomized order, and blank samples were injected regularly and used to assess carryover and exclude background signals. Following data pre-processing, statistical analysis was performed by univariate and multivariate analysis.

**9. Analysis and quantification of cell death**

HCT116 cells were stimulated with rafoxanide (2.5 μM) or vehicle (DMSO) for 15, 30, and 60 minutes. Cells were harvested and stained with Hoechst 33342, YO-PRO-1, and PI dyes for 30 minutes on ice to detect chromatin condensation, apoptosis or necrotic cells, respectively (#V23201, Invitrogen). Cells were then washed with PBS and analyzed by flow cytometry.
